# *GLIPR1* expression is reduced in multiple myeloma but is not a tumour suppressor in mice

**DOI:** 10.1371/journal.pone.0228408

**Published:** 2020-01-29

**Authors:** Natasha Friend, Jacqueline E. Noll, Khatora S. Opperman, Kimberley C. Clark, Krzysztof M. Mrozik, Kate Vandyke, Duncan R. Hewett, Andrew C. W. Zannettino

**Affiliations:** 1 Adelaide Medical School, Faculty of Health and Medical Sciences, University of Adelaide, Adelaide, Australia; 2 Cancer Program, Precision Medicine Theme, South Australian Health and Medical Research Institute, Adelaide, Australia; University of Catanzaro, ITALY

## Abstract

Multiple myeloma, a plasma cell malignancy, is a genetically heterogeneous disease and the genetic factors that contribute to its development and progression remain to be fully elucidated. The tumour suppressor gene *GLIPR1* has previously been shown to be deleted in approximately 10% of myeloma patients, to inhibit the development of plasma cell tumours in ageing mice and to have reduced expression levels in the plasma cells of patients with light-chain amyloidosis, a myeloma-related malignancy. Therefore, we hypothesised that *GLIPR1* may have tumour suppressor activity in multiple myeloma. In this study, we demonstrate that plasma cell expression of *GLIPR1* is reduced in the majority of myeloma patients and *Glipr1* expression is lost in the 5TGM1 murine myeloma cell line. However, overexpression of GLIPR1 in a human myeloma cell line did not affect cell proliferation *in vitro*. Similarly, re-expression of Glipr1 in 5TGM1 cells did not significantly reduce their *in vitro* proliferation or *in vivo* growth in C57BL/KaLwRij mice. In addition, using CRISPR-Cas9 genome editing, we generated C57BL/*Glipr1*^-/-^ mice and showed that loss of Glipr1 *in vivo* did not affect normal haematopoiesis or the development of monoclonal plasma cell expansions in these mice up to one year of age. Taken together, our results suggest that *GLIPR1* is unlikely to be a potent tumour suppressor in multiple myeloma. However, it remains possible that the down-regulation of *GLIPR1* may cooperate with other genetic lesions to promote the development of myeloma.

## Background

Multiple myeloma (MM) is a haematological malignancy characterised by the uncontrolled proliferation of antibody-producing plasma cells (PCs) within the bone marrow (BM). MM is defined by the presence of 10% or more clonal PCs in the BM and one or more myeloma-defining event(s) [1]. Myeloma-defining events include evidence of end-organ damage, such as hypercalcemia, renal insufficiency, anaemia, and bone lesions. The MM PCs produce large amounts of a non-functional monoclonal immunoglobulin (paraprotein/M protein) that can be detected in blood serum and/or urine [2]. Almost all MM cases are preceded by the premalignant condition monoclonal gammopathy of undetermined significance (MGUS), a benign clonal PC proliferation characterised by less than 10% PCs in the BM and the absence of end-organ damage [3, 4]. Approximately 3–4% of people over 50 years of age have MGUS and they are at risk of progressing to MM at a rate of 1% per year, although the time to progression is variable [5]. While recent treatment advances have improved the median overall survival for MM to ~6 years [6], the majority of patients relapse with refractory disease and thus MM remains a largely incurable disease [7].

The development of MGUS is initiated in a post-germinal centre B cell by a primary cytogenetic event; namely hyperdiploidy or a chromosomal translocation involving the immunoglobulin heavy-chain gene [8]. Malignant transformation and MM disease progression is believed to occur due to the accumulation of secondary “genetic hits”, including further chromosomal rearrangements and DNA mutations, as well as transcriptional and epigenetic changes [8]. Recent studies have revealed that there is significant interpatient genetic heterogeneity in MM, with low recurrence rates for many mutations [9–13]. In addition, there is considerable intrapatient genetic heterogeneity, with the majority of MM patients displaying a complex subclonal architecture that is dynamic and can evolve over time [10, 11, 14–20]. Notably, many of the chromosomal abnormalities and genetic lesions identified in MM PCs are also found at the MGUS stage [16, 19, 21], which highlights the possibility that novel epigenetic changes and/or PC-extrinsic factors are involved in driving the progression from asymptomatic MGUS to malignant MM.

*GLIPR1* is a ubiquitously expressed gene that encodes a member of the cysteine-rich secretory proteins, antigen 5, and pathogenesis-related 1 proteins (CAP) superfamily with unspecified function [22, 23]. A reduction in *GLIPR1* expression was shown to be a feature of several solid cancers, including prostate, lung and bladder cancer, as well as sarcoma [24–27]. In all cases, *GLIPR1* demonstrated tumour suppressor activity *in vitro* [24–29], and, furthermore, *GLIPR1* expression was shown to suppress prostate cancer tumour growth i*n vivo* [30]. GLIPR1 has been found to mediate its tumour suppressor effects in prostate cancer cells through several different mechanisms [29, 31, 32]. Specifically, GLIPR1 was shown to promote apoptosis by increasing reactive oxygen species production [29] and by modulating the regulation of apoptosis-related gene expression by HSC70 [31]. Interestingly, GLIPR1 was found to cause cell cycle arrest by decreasing expression of the oncogenic *MYC* transcription factor [32], which is commonly up-regulated in MM [33–35] and a proven driver of MM development [36].

The putative tumour suppressor role of GLIPR1 is further supported by the finding that *Glipr1*^*-/-*^ mice had reduced survival due to increased rates of spontaneous malignancy, although tumour development was of late onset (after ~500 days) and incomplete penetrance (~40%) [29]. Notably, 40% of the tumours in the *Glipr1*^*-/-*^ mice were classified as plasmacytomas, a localised PC malignancy that frequently progresses to MM [37, 38]. In addition, the same study found that the expression levels of *GLIPR1* were significantly reduced in the human MM cell lines U266 and RPMI-8226 compared to normal B cells [29]. Furthermore, hemizygous chromosomal deletions encompassing *GLIPR1* have been reported in PCs from 9.4% of MM patients [39], and down-regulation of *GLIPR1* was one of only 38 gene expression changes identified in PCs from patients with the MM-related malignancy light-chain amyloidosis, when compared with normal PCs [40]. Together, these data support a potential tumour suppressor role for *GLIPR1* in MM.

In this study, we show that *GLIPR1* expression is reduced in MM patient–derived PCs compared to PCs isolated from healthy controls. In addition, we demonstrate that *Glipr1* expression is lost in the C57BL/KaLwRij-5TGM1 murine model of MM and assess the impact of its reintroduction on tumour growth *in vivo*. Furthermore, we generate C57BL/*Glipr1*^-/-^ mice and investigate the effect of *Glipr1* loss on the development of PC abnormalities for up to 12 months.

## Methods

### Ethics statement

All procedures involving animals in this study were approved by the South Australian Health and Medical Research Institute's Animal Ethics Committee. A total of n = 26 C57BL/6 mice, n = 31 C57BL/KaLwRij mice and n = 24 C57BL/*Glipr1*^*-/-*^ mice were used in this study. All animals were housed in individually ventilated cages in specific pathogen free conditions and had continuous access to standard food, water and environmental enrichment. All of the mice in this study were euthanised by CO_2_ inhalation.

### Publicly available microarray data

For analysis of *GLIPR1* expression in CD138-selected BM PCs from newly diagnosed MGUS or MM patients or healthy controls, two independent microarray datasets were used: E-GEOD-16122 (normal, n = 5; MGUS, n = 11; MM, n = 133; [41]) and E-GEOD-6477 (normal, n = 15; MGUS, n = 22; MM, n = 69; [42]). Analysis of *GLIPR1* expression in different gene expression-defined (UAMS) molecular subsets of newly diagnosed MM patients was conducted using microarray dataset GSE4581 (n = 414; [43]). Analysis of overall survival in MM patients stratified on the basis of median *GLIPR1* expression in CD138^+^ BM PCs at diagnosis was carried out using the dataset E-TABM-1138 (n = 142; [44]). Correlative analysis of *GLIPR1* and *MYC* gene expression in CD138-selected BM PCs from newly diagnosed MM patients was performed using E-GEOD-6477, GSE4581 and E-GEOD-16122. GSE4581 and E-TABM-1138 were conducted on Affymetrix GeneChip Human Genome U133 plus 2.0 arrays, while E-GEOD-16122 and E- GEOD-6477 were conducted on U133A arrays. Processed microarray data for GSE4581, E-GEOD-6477 and E-GEOD-16122 were downloaded from ArrayExpress (EMBL-EBI) or Gene Expression Omnibus (NCBI) and were log_2_ transformed where required. For E-TABM-1138, raw microarray data (CEL files) were downloaded from ArrayExpress, normalised by RMA using the Bioconductor package affy and R (v3.03) and log_2_ transformed.

### Cell culture

All cell lines were maintained in a humidified environment at 37°C in the presence of 5% CO_2_ and were manipulated within a class II biological safety cabinet. Unless otherwise specified, all cell culture reagents were sourced from Sigma-Aldrich and all media were supplemented with 2 mM L-glutamine, 100 U/mL penicillin, 100 μg/mL streptomycin, 1 mM sodium pyruvate, and 10 mM HEPES buffer. All cell lines were tested for mycoplasma infection using a MycoAlert^TM^ Mycoplasma Detection Kit (Lonza) prior to use and were maintained in culture for a maximum of 4 weeks. Human myeloma cell lines (HMCLs) RPMI-8226, LP-1, and U266 were obtained from the ATCC between 2000 and 2003; STR authentication was not conducted on these lines as they were obtained directly from the ATCC. HMCLs OPM2, H929 and JIM-1 were provided by Prof. Andrew Spencer (Monash University, Melbourne, Australia); the OPM2 and H929 cell lines were authenticated using STR analysis (no STR reference for JIM-1) performed by the Molecular Genetics Laboratory, SA Pathology, using an AmpFLSTR Identifiler PCR Amplification Kit (Thermo Fisher Scientific). HMCLs were maintained in RPMI-1640 medium with 10% fetal calf serum (FCS; Thermo Fisher Scientific). The murine MM 5TGM1 PC line was originally kindly provided by Assoc Prof Claire Edwards (University of Oxford, UK). 5TGM1 cells expressing both green fluorescent protein (GFP) and luciferase were previously generated using the retroviral expression vector NES‐TGL [45]. 5TGM1 cells were maintained in Iscove’s Modified Dulbecco’s Medium (IMDM) with 20% FCS. The mouse BM stromal cell (BMSC) line OP9 was obtained from the ATCC and was maintained in Dulbecco's Modified Eagle Medium (DMEM) with 10% FCS.

### Quantitative reverse transcription polymerase chain reaction (RT-qPCR)

Total RNA was isolated from cells using TRIzol^TM^ Reagent (Thermo Fisher Scientific) according to the manufacturer’s instructions, unless otherwise specified. For mouse and human CD138^+^ PCs, RNA was reverse transcribed into cDNA using Sensiscript (Qiagen). For all other tissues and cell lines, RNA (2 μg) was reverse transcribed into cDNA using SuperScript^TM^ IV (Thermo Fisher Scientific) according to the manufacturers’ instructions. Real-time polymerase chain reaction (PCR) was conducted using 1x RT^2^ SYBR^®^ Green qPCR Mastermix (QIAGEN) and the following primer sequences on the CFX Connect^TM^ Real-Time PCR Detection System (Bio-Rad): human *GLIPR1* (F: 5’-TCACTGGGAGAGAACATCTGGA-3’ R: 5’-GGAAAGAGCGTCAAAGCCAG-3’), human *GAPDH* (F: 5’-ACCCAGAAGACTGTGGATGG-3’ and R: 5’-CAGTGAGCTTCCCGTTCAG-3’), mouse *Glipr1* (F: 5’-AGGTTGTTTGGGCAGACAGT-3’ and R: 5’-TTTTGGGCAATCACTGCACG-3’), mouse *Myc* (F: 5’-TCGAGCTGTTTGAAGGCTGG-3’ and R: 5’-ACGGAGTCGTAGTCGAGGTC-3’) and mouse/human *Actb/ACTB* (F: 5′-TTGCTGACAGGATGCAGAAG-3′ and R: 5′-AAGGGTGTAAAACGCAGCTC-3′). Changes in gene expression were calculated relative to *GAPDH* or *Actb* using the $2^{{-\Delta \Delta C}_{t}}$ method [46].

### Generation of Glipr1/GLIPR1-overexpressing cell lines

A 5TGM1 cell line overexpressing *Glipr1* was generated by infection with a pRUFimCH2 retroviral vector [47] harbouring a full-length cDNA encoding murine *Glipr1*, which was amplified from C57BL/6 thymus-derived cDNA by PCR. A H929 HMCL overexpressing *GLIPR1* was generated by infection with a LeGOiT2 lentiviral vector [48] harbouring a full-length cDNA encoding human *GLIPR1*, which was amplified by PCR from HMCL LP-1 cDNA. Briefly, following sequence verification, the *Glipr1*/*GLIPR1* viral vectors were transfected into HEK-293T cells and viral particle-containing supernatant was used to infect 5TGM1 or H929 cells. Cells were subjected to fluorescence activated cell sorting (FACS) for mCherry/Tomato protein expression on a FACSAria^TM^ Fusion (BD Biosciences) and pooled *Glipr1*/*GLIPR1*-overexpressing cell lines and empty vector (EV) controls were established. The basal luciferase activity of 5TGM1-Glipr1 and 5TGM1-EV control cells was assessed by seeding an equal number of cells in quadruplicate in a 96-well plate, adding 0.3 mg/mL D-luciferin (Biosynth) and performing bioluminescence imaging using the IVIS^®^ Spectrum (PerkinElmer). No significant difference in luciferase activity was observed between the two modified 5TGM1 cell lines.

### Western blot analysis

Cells were lysed using radioimmunoprecipitation assay (RIPA) buffer [1% NP-40 (v/v), 20 mM HEPES, 150 mM NaCl, 10% glycerol (v/v), 2 mM Na_3_VO_4_, 10 mM Na_4_P_2_O_7_, 2 mM NaF, and 1x cOmplete^TM^ EDTA-free Protease Inhibitor Cocktail (Roche)]. The protein concentration in each cell lysate was determined using the RC DC^TM^ Protein Assay Kit (Bio-Rad), according to manufacturer’s instructions. Equal concentrations of total protein were separated by SDS-PAGE using the Mini-PROTEAN^TM^ III System (Bio-Rad). Proteins were then transferred from the gel to a nitrocellulose 0.45 μm membrane (Bio-Rad) using the Mini Trans-Blot® Electrophoretic Transfer Cell (Bio-Rad). Membranes were probed with specific antibodies against mouse Glipr1 (1:250; #AF4468, R&D Systems), human GLIPR1 (1:500; #H00011010-A01, Abnova) and Hsp90/HSP90 (1:2,500; #7947, Santa Cruz Biotechnology). These were then visualised using DyLight-680/800-conjugated secondary antibodies (1:10,000; Thermo Fisher Scientific) and an Odyssey® CLx Imager (LI-COR). Densitometry was performed using ImageJ software (http://fiji.sc).

### Proliferation assays

The proliferation of H929 cells *in vitro* was assessed by WST-1 assay. H929 cells were plated at 1x10^5^ cells/mL in triplicate in RPMI-1640 medium containing 10% FCS (100 μL per well) using four replicate 96-well plates. The plates were incubated at 37°C with 5% CO_2_ and every 24 hours from day 0 to 3, 10 μL of WST-1 Reagent (Roche) was added to the wells of one plate. Following a two-hour incubation, the absorbance of each well at 450 nm was measured using the iMark^TM^ Microplate Absorbance Reader (Bio-Rad) and the plate discarded. The background from medium-only wells was subtracted from the absorbance values and the fold-change in absorbance for each cell line on days 1–3 was calculated relative to day 0.

The proliferation of 5TGM1 cells *in vitro* was assessed by measuring luciferase activity. 5TGM1 cells were seeded in triplicate at 1 × 10^5^ cells/mL in IMDM containing 20% FCS with, or without, a confluent layer of OP9 cells. After three days, the 5TGM1 cells were enumerated by measuring luciferase activity. Briefly, cells were collected with the aid of trypsin, washed in PBS and lysed in 40 μL of 1x Luciferase Cell Culture Lysis Reagent (Promega). The lysates (20 μL) were transferred into an opaque 96-well plate and 100 μL of luciferase reaction buffer [5 mM MgCl_2_, 30 mM HEPES, 150 μg/mL D-luciferin (Biosynth) and 150 μM ATP] was added per well immediately prior to reading the bioluminescence signal on a luminometer (Wallac 3000).

### Purification of primary murine PCs and RNA isolation

C57BL/6 and KaLwRij mouse BM was collected from cleaned femora and tibiae by repeatedly flushing the bones with 5 mL of chilled PFE buffer (PBS, 2%FCS, 2 mM EDTA) using a 10 mL syringe and 21 G needle. The BM cells were stained with a rat anti-CD138 primary antibody (#300506, R&D Systems) and an anti-rat IgG-PE secondary antibody (#3030–09, Southern Biotech), followed by FACS for PE^+^ cells using the FACSAria^TM^ Fusion (BD Biosciences). Total RNA from primary murine PCs was subsequently isolated using the All Prep DNA/RNA Micro Kit (Qiagen), according to the manufacturer’s instructions.

### Reverse transcription polymerase chain reaction (RT-PCR)

Total RNA was extracted from isolated murine PCs/5TGM1 cells and reverse transcribed as described above. Separate PCRs for *Glipr1* and *Actb* (see primer sequences above) was performed using AmpliTaq Gold^TM^ DNA Polymerase (Thermo Fisher Scientific) on a VeritiTM Thermal Cycler (Thermo Fisher Scientific). The PCR products from the cDNA were then visualised by agarose gel electrophoresis using a 2% (w/v) agarose gel containing 1:10,000 GelRed® (Biotium).

### Colony formation assay

5TGM1 cells were seeded (200 cells per 35 mm dish) in duplicate in MethoCultTM semi-solid methylcellulose medium (StemCell Technologies), according to the manufacturer’s instructions. After 12 days of culture at 37°C and 5% CO_2_, colonies (> 50 cells) were manually counted using a light microscope.

### C57BL/KaLwRij-5TGM1 murine model of MM

C57BL/KaLwRijHsd (KaLwRij) mice were originally kindly provided by Prof Andrew Spencer (Monash University, Australia) and were rederived, bred and housed at the South Australian Health and Medical Research Institute Bioresources Facility. Age- and sex-matched 6-8-week-old KaLwRij mice were injected intravenously with 5 × 10^5^ 5TGM1-Glipr1 or 5TGM1-EV cells in 100 μL of sterile PBS. For each cell line, 4–5 mice were injected in three replicate experiments, resulting in a total of n = 14–15 mice per experimental group. Post-tumour cell administration, animals were observed daily for their level of activity/mobility; normal eating and drinking; and grooming behaviour and appearance. In addition, the animals were weighed three times per week. At the first signs of distress (e.g. scruffiness, hunching, reluctance to move), hind limb paralysis, signs of terminal illness/bleeding or >10% weight loss an animal was removed from the study and euthanised by CO_2_ inhalation. Tumour development was monitored weekly by *in vivo* bioluminescence imaging, as previously described [47]. Animals were monitored daily for any adverse effects and were euthanised at the first signs of morbidity. Based on our previous experience with parental 5TGM1-BMx1 cells in this model (mean BLI at wk 4: 1.10E+08, STDEV: 5.20E+07), 14 animals per group provides sufficient power to detect a 50% decrease in tumour burden by a two-sided statistical test, with alpha: 0.05 and power: 0.80. At experimental endpoints, topical emla 5% anaesthetic cream (lignocaine and prilocaine) was applied to the tail and peripheral blood serum was isolated by a tail bleed. Serum protein electrophoresis (SPEP) was then performed using the Hydragel Protein(E) Kit (Sebia), according to the manufacturer’s instructions. The intensity of the paraprotein band/M-spike was quantitated and normalised to the albumin band using Image Lab Software v6.0.1 (Bio-Rad). The presence of an M-spike on the SPEP gel for the 12-month-old mice was assessed by densitometry using ImageJ software (http://fiji.sc).

### Generating *Glipr1* knockout mice

C57BL/*Glipr1*^-/-^ (*Glipr1*^-/-^) mice were generated by the South Australian Genome Editing Facility (University of Adelaide, Australia) using CRISPR-Cas9. Briefly, gRNAs were designed that flanked the first exon of *Glipr1* (gRNA 1: 5’-ATTGGTTCTTGCCAAATGGGC-3’ and gRNA 2: 5’-ATCAGCGGCTCTCGACCCGT-3’). These gRNAs and Cas9 mRNA were injected into C57BL/6 zygotes, which were then transferred to pseudopregnant recipients. Founder pups were genotyped by PCR using separate reactions to detect wildtype (WT) alleles (P1: 5’-TTGCATATTAGCCCTCAGAACCCTTAGT-3’ and P3: 5’-TGTGTGCCTTTGTCTGAGGTC-3’) and deletion alleles (P1 and P2: 5’-ACACGGTAGCTTTTGTATGAAGGAACAGT-3’) of *Glipr1*. PCR products from potential deletion alleles were Sanger sequenced. A male founder that harboured a *Glipr1* exon 1 deletion was crossed with C57BL/6 (WT) mice and the resultant *Glipr1* deletion heterozygotes (*Glipr1*^+/-^) were intercrossed. The progeny that were homozygous for the *Glipr1* deletion were then incrossed to generate a stock *Glipr1*^-/-^ colony. The first litter of *Glipr1*^-/-^ mice underwent standard early life physical and behavioural checks at days 1–6, day 14, week 3 and week 6. Daily checks were performed for cages as a whole and there were no *Glipr1*^-/-^ mice that exhibited illness or distress. The *Glipr1*^-/-^ mice and the WT mice used in comparative analyses were from separate colonies and were not littermates.

### HEMAVET analysis

Peripheral blood samples were collected from mice by a tail bleed (emla topical anaesthetic cream applied) into EDTA-coated microvette tubes (Sarstedt). Complete blood counts were performed using a HEMAVET950 automated blood analyser (Drew Scientific), according to the manufacturer’s instructions.

### Flow cytometric cell lineage analysis

For analysis of 12-week-old mice, a total of n = 12 C57BL/6 mice and n = 12 C57BL/*Glipr1*^*-/-*^ mice were used in three independent experiments. For analysis of 12-month old mice, a total of n = 10 C57BL/6 mice and n = 10 C57BL/*Glipr1*^*-/-*^ mice were used in one experiment. BM cells were extracted from long bones (femora and tibiae) using a mortar and pestle and spleen cells were collected by pushing the excised and cleaned tissue through a 70 μm filter using a syringe plunger. Cells were stained with Fixable Viability Stain 700 (BD Biosciences) according to the manufacturer’s instructions and blocked with mouse gamma globulin (1:100; Jackson ImmunoResearch). For detection of B cells/PCs in the BM and spleen, cells were stained with B220-FITC (RA3-6B2, BioLegend), IgM-PE-Cy7 (R6-60.2, BD Biosciences) and CD138-BV421 (281–2, BD Biosciences). For detection of BM monocytes/macrophages and granulocytes in the BM, cells were stained with CD11b-APC-Cy7 (M1/70, BD Biosciences), CD169-PE (3D6.112, BioLegend), Ly6G-PE-Cy7 (1A8, BioLegend) and F4/80-Pacific Blue (Cl:A3-1, Bio-Rad). For the detection of hematopoietic stem cells (HSCs) and endothelial cells in the BM, mature Lin^+^ cells were excluded by incubation with a lineage cocktail of biotin-conjugated antibodies [B220, CD3, CD4, CD5, CD8, Gr1, Ter119 (BioLegend)] followed by streptavidin-PE (SouthernBiotech) secondary. For quantitating HSCs, cells were concurrently stained with Sca-BV786 (D7, BD Biosciences), CD117-PE-Cy7 (2B8, BD Biosciences), CD135-PE-CF594 (A2F10.1, BD Biosciences), and CD34-BV421 (RAM34, BD Biosciences). For detection of BM endothelial cells, cells were concurrently stained with Sca-BV786, CD31-BV421 (390, BioLegend), CD144-PE-Cy7 (BV13, BioLegend) and CD45.2-BUV395 (104, BD Biosciences). Mesenchymal stem cells (MSCs) were quantitated from compact bone preparations as previously described [49]. To assist with compensation and gating cell populations, unstained cells, single-stained CompBeads (BD Biosciences) and fluorescence minus one (FMO) stained cells were prepared and analysed as controls for every panel. Cells were fixed in 1% neutral buffered formalin, 2% glucose, and 0.01% sodium azide in PBS and subsequently analysed on a LSRFortessa^TM^ X-20 flow cytometer using FACSDiva^TM^ software v8.0 (BD Biosciences). Analysis of flow cytometry data, including compensation, was performed using Flowjo software (Treestar).

### Murine B cell *ex vivo* proliferation assay

Single splenic cell suspensions from age- and sex-matched 12-week-old C57BL/6 WT mice and *Glipr1*^-/-^ mice (n = 3 mice per genotype in three replicate experiments) were generated, as described above. Red blood cells were lysed by incubating once with red blood cell lysis buffer (150 mM NH_4_Cl, 10 mM KHCO_3_ and 0.1 mM EDTA, pH 8.0) for 10 minutes and the remaining cells were washed with PFE buffer. Resting B cells were isolated by MACS negative selection using a mouse B Cell Isolation Kit (Miltenyi Biotec), according to the manufacturer’s instructions. Successful depletion of CD43^+^ cells, was confirmed by staining the cells with CD43-PE (eBioR2/60, Thermo Fisher Scientific) and B220-FITC and analysing by flow cytometry, as described above. Purified resting B cells were resuspended in RPMI-1640 medium with 10% FCS, standard supplements, 50 nM 2-Mercaptoethanol and 1x MEM Non-Essential Amino Acids Solution. The cells (1 x 10^5^ cells per well) were seeded in triplicate in 100 μL of medium with a final concentration of 20 ng/mL IL-4 and 20 μg/mL LPS using duplicate 96-well plates. One plate immediately underwent a WST-1 assay, according to the manufacturer’s instructions. The second plate was incubated at 37°C with 5% CO_2_ for 3 days prior to performing the WST-1 assay. The absorbance of each well at 450 nm was measured using the iMark^TM^ Microplate Absorbance Reader (Bio-Rad). The medium-only background was subtracted from the absorbance values and the fold-change in absorbance was calculated.

### Statistical analysis

Unless otherwise described, statistical analysis was performed using GraphPad Prism v8.0.0 (GraphPad Software). When two groups were being compared for a single variable, a parametric paired t test, a parametric unpaired t test or a non-parametric Mann-Whitney U test was used. For the parametric tests a normal Gaussian distribution was assumed. When three or more patient groups were being compared for a single variable, a non-parametric Kruskal-Wallis test with Dunn’s multiple comparisons test was used. For time-course experiments, groups were compared using a two-way ANOVA with Sidak’s multiple comparisons test. Correlations were assessed using Pearson correlation coefficients. Overall survival was assessed using Kaplan–Meier curves; comparisons between groups were made using the logrank (Mantel–Cox) test and the Mantel–Haenszel hazard ratio. Differences were considered statistically significant when *P* < 0.05.

## Results

### *GLIPR1* expression is reduced in the PCs of MM patients

**Fig 1:**
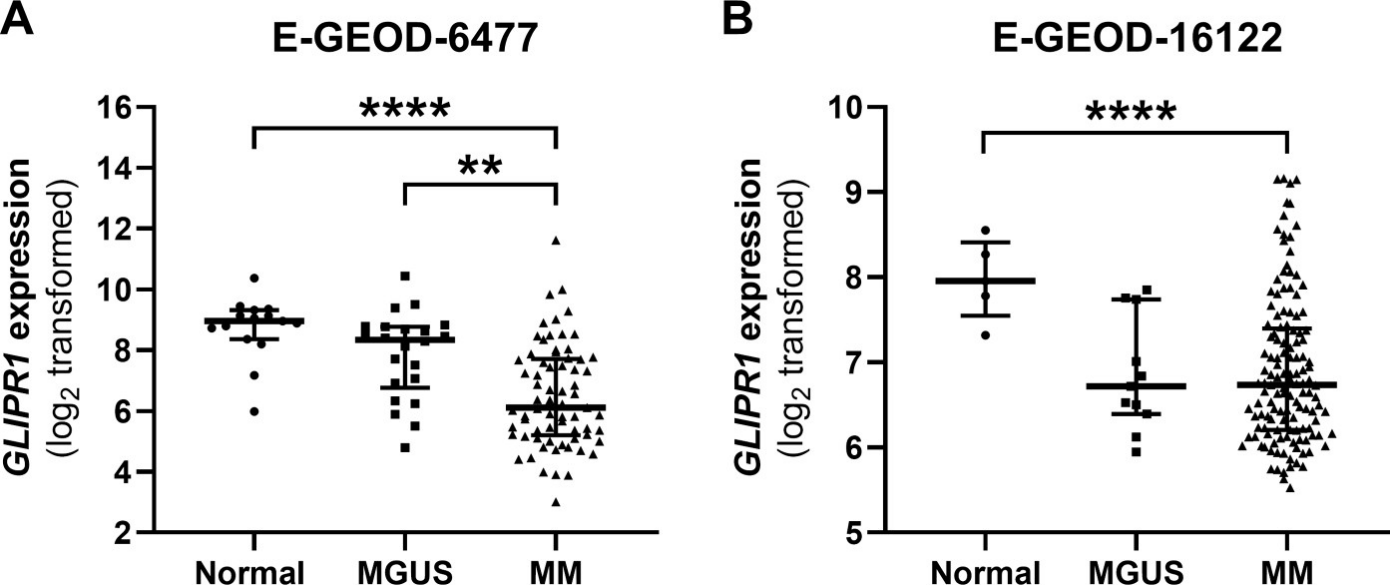
*GLIPR1* mRNA expression is down-regulated in PCs from MM patients. *In silico* analysis was performed on publicly available datasets analysing gene expression in CD138^+^ PCs isolated from MGUS (n = 22) and MM (n = 69) patients and healthy controls (n = 15; E-GEOD-6477; **A**) and MGUS (n = 11) and MM (n = 133) patients and healthy controls (n = 5; E-GEOD-16122; **B**). Scatter dot plots show median and interquartile range. ****P < 0.0001, **P < 0.01; Kruskal-Wallis test with Dunn’s multiple comparisons test.

Given that the *GLIPR1* tumour suppressor gene was found to have significantly reduced expression levels in PCs from patients with amyloidosis [40] and to be deleted in 9.4% of MM patients [39], we hypothesised that the expression of *GLIPR1* may also be down-regulated in the malignant PCs of MM patients. To assess this, *in silico* analysis of *GLIPR1* expression in purified PCs from newly diagnosed patients with MGUS or MM and healthy controls was performed using two independent, publicly available microarray datasets. Significantly reduced expression of *GLIPR1* was observed in PCs from MM patients when compared to PCs from healthy donors in both dataset E-GEOD-6477 (*P* < 0.0001; Fig 1A) and dataset E-GEOD-16122 (*P* = 0.022; Fig 1B). Reduced *GLIPR1* expression, defined as less than the lower 95% confidence interval for *GLIPR1* expression in the healthy cohort, was observed in 84% (58/69; E-GEOD-6477) and 75% (100/133; E-GEOD-16122) of MM patients. In addition, *GLIPR*1 mRNA expression was significantly reduced in the PCs of MM compared with MGUS patients in E-GEOD-6477 (*P* = 0.003; Fig 1A), whereas there was no difference in E-GEOD-16122 (*P* > 0.999; Fig 1B).

To assess whether reduced *GLIPR1* expression is associated with primary genetic events in MM patients, the publicly available microarray dataset GSE4581 was partitioned into gene expression profiling-defined molecular subgroups (UAMS classification [43]) and *GLIPR1* levels were compared. *GLIPR1* expression was reduced in the hyperdiploid (HY) and the reduced lytic bone disease (LB) subgroups compared to the MAF (MF) or MMSET (MS) translocation subgroups (*P* < 0.01; S1A Fig). *GLIPR1* levels were also reduced in the HY subgroup compared to the chromosomal translocations involving cyclin D1 and cyclin D3 (CD2) subgroup (*P* < 0.01; S1A Fig). In addition, to assess the potential prognostic significance of *GLIPR1* expression, the overall survival of newly diagnosed MM patients from publicly available microarray dataset E-TABM-1138 (n = 142) was analysed. When patients were stratified into two groups based on median *GLIPR1* expression, there was no significant difference in overall survival between MM patients with below median (low) *GLIPR1* expression and those patients with above median (high) *GLIPR1* levels (*P* = 0.760; S1B Fig). Furthermore, given that GLIPR1 was shown to down-regulate MYC in prostate cancer [32], the relationship between *GLIPR1* expression and *MYC* expression in MM PCs from newly diagnosed patients was also assessed *in silico*. The expression of *GLIPR1* was found to be negatively correlated with *MYC* expression in three independent microarray datasets (E-GEOD-6477: r = -0.349, *P* = 0.0033; E-GEOD-16122: r = -0.199, *P* = 0.0214; GSE4581: r = -0.221, *P* < 0.0001; S1C–S1E Fig). The prognostic significance of MYC expression in the microarray dataset E-TABM-1138 was also analysed. There was no statistical difference in the overall survival for patients with above median *MYC* expression versus patients with below median *MYC* expression (S1F Fig).

### *GLIPR1* overexpression does not affect HMCL proliferation *in vitro*

**Fig 2:**
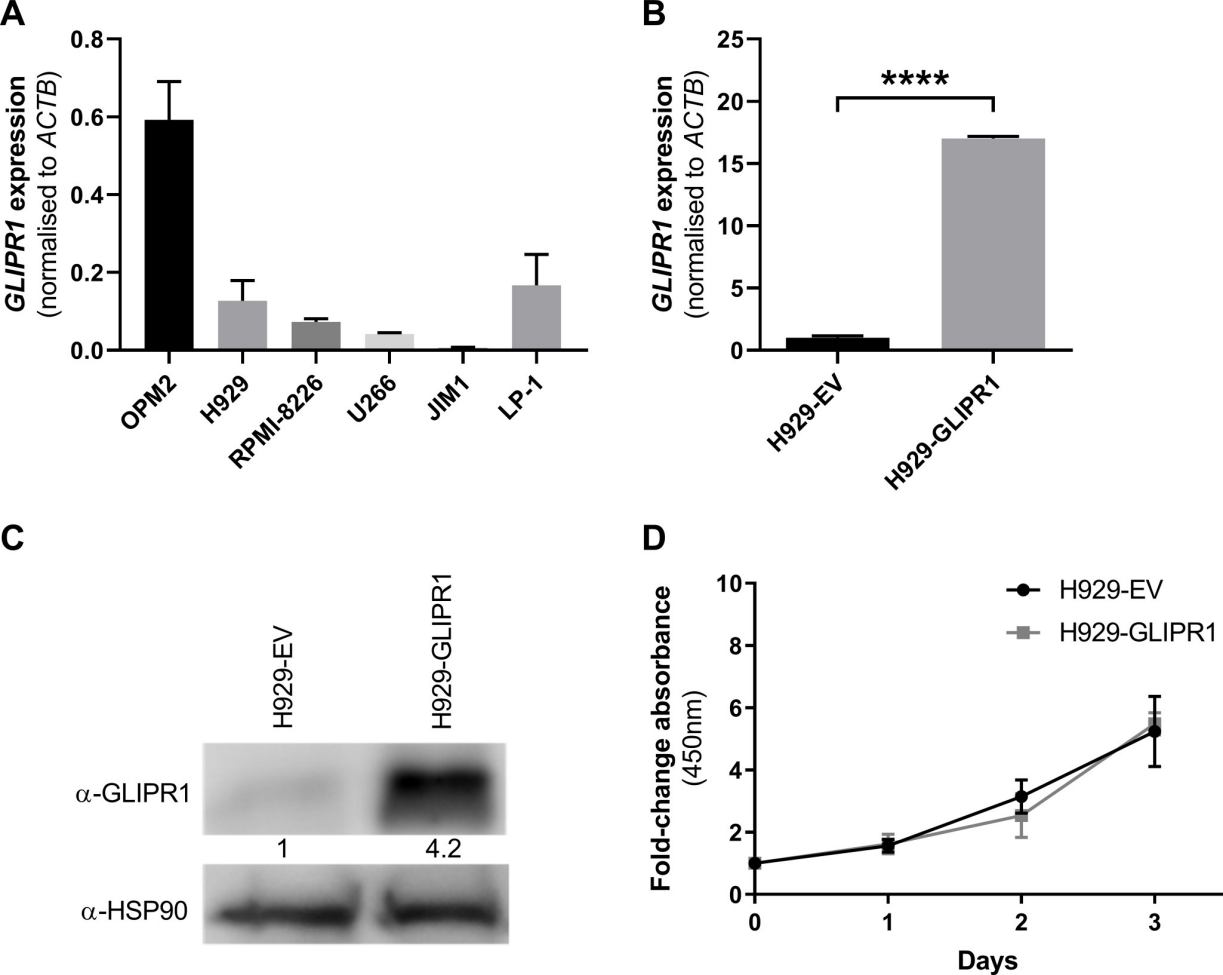
*GLIPR1* overexpression does not affect HMCL proliferation *in vitro*. (**A**) The expression levels of *GLIPR1* mRNA in six HMCLs were assessed by RT-qPCR (normalised to *ACTB*). Graph depicts the mean + SD of triplicates. (**B**) RT-qPCR for *GLIPR1* mRNA was performed on RNA from H929-EV cells and H929-GLIPR1 cells. *GLIPR1* expression levels were normalised to *ACTB* and were expressed relative to H929-EV cells. Graph depicts the mean + SD of triplicates. ****P < 0.0001, unpaired t test. (**C**) The expression of GLIPR1 protein in H929-EV cells and H929-GLIPR1 cells was assessed by Western blot. HSP90 was used as the loading control. (**D**) The basal proliferation of H929-EV and H929-GLIPR1 cells was assessed over 3 days by WST-1 assay. Graph depicts the mean ± SD of n = 3 independent experiments.

The expression of *GLIPR1* in a panel of HMCLs was assessed and low *GLIPR1* mRNA levels were observed in five of the six HMCLs examined by RT-qPCR (Fig 2A). To determine whether *GLIPR1* expression levels affect the growth of HMCLs *in vitro*, H929 cells were transduced with a *GLIPR1* expression construct (H929-GLIPR1) or empty vector control (H929-EV). The overexpression of *GLIPR1* in the H929-GLIPR1 cells was confirmed by RT-qPCR (Fig 2B) and Western blot (Fig 2C). The proliferation of the H929-GLIPR1 cells was compared to that of the H929-EV cells by a WST-1 assay. No significant difference in basal growth was observed between the cell lines over three days (*P* = 0.794; Fig 2D).

### *Glipr1* is not expressed in 5TGM1 murine MM cells but re-expression does not affect tumour cell proliferation *in vitro*

**Fig 3:**
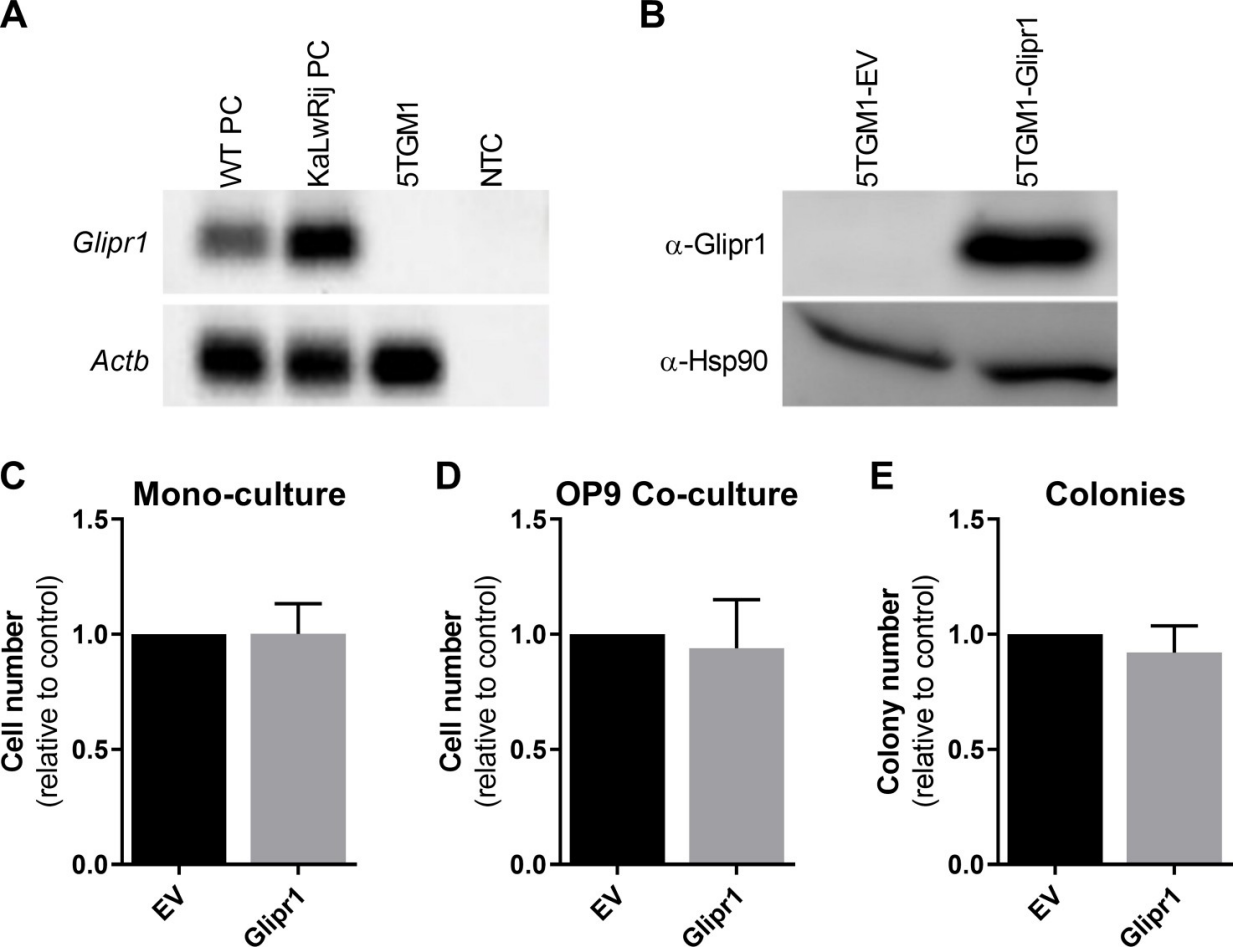
*Glipr1* expression is lost in 5TGM1 cells but its re-expression does not affect cell proliferation *in vitro*. (**A**) The expression of *Glipr1* mRNA was assessed by RT-PCR in CD138^+^ PCs from healthy WT (C57BL/6) mice and KaLwRij mice, as well as the KaLwRij-derived 5TGM1 MM PC line. The products were run on a 2% agarose gel and stained with GelRed. *Actb* was used as a positive control. NTC = no template control. (**B**) The expression of Glipr1 protein in 5TGM1-Glipr1 (Glipr1) and control 5TGM1-EV (EV) cells was assessed by Western blot. Hsp90 was used as the loading control. The number of 5TGM1-Glipr1 cells in mono-culture (**C**) or co-culture with OP9 bone marrow stromal cells (**D**) was assessed by measuring luciferase activity after 3 days. Cell number is expressed relative to the EV control cells. (**E**) Colony formation by 5TGM1-Glipr1 cells versus 5TGM1-EV cells was assessed in semi-solid methylcellulose-containing medium after 12 days. Colony number is expressed relative to the EV control cells. Graphs depict the mean + SD of n = 3 independent experiments.

To investigate the potential role of *Glipr1* in suppressing MM tumour development, the widely studied C57BL/KaLwRij (KaLwRij)-5TGM1 murine model of MM was utilised [50–52]. In this model, the 5TGM1 murine MM cell line, derived from a spontaneous PC tumour in an aged KaLwRij mouse, is inoculated intravenously into young syngeneic KaLwRij mice. This results in the development of disease that recapitulates many features of human MM, including PC tumour growth in the BM, paraprotein production and lytic bone disease [50–52]. Firstly, the expression of *Glipr1* was assessed in purified PCs from healthy wildtype (WT) C57BL/6 mice and KaLwRij mice, as well as the KaLwRij-derived 5TGM1 MM cell line. While *Glipr1* mRNA expression was detected in normal PCs from both the WT and KaLwRij mouse strains, *Glipr1* expression was undetectable in the 5TGM1 MM PC line (Fig 3A), consistent with *Glipr1* being a tumour suppressor.

To determine whether *Glipr1* has tumour suppressor activity in the KaLwRij-5TGM1 MM mouse model, 5TGM1 cells were transduced with a *Glipr1* expression construct (5TGM1-Glipr1) or empty vector control (5TGM1-EV). The re-expression of *Glipr1* in the 5TGM1-Glipr1 cells was confirmed by Western blot (Fig 3B). Given that enforced GLIPR1 expression in prostate cancer cells caused a reduction in *MYC* mRNA levels [32], the effect of Glipr1 re-expression on the expression levels of *Myc* in 5TGM1 cells was assessed. Using RT-qPCR, no difference in *Myc* mRNA expression was observed between 5TGM1-Glipr1 and 5TGM1-EV cells (*P* = 0.799, S2 Fig). In addition, *Glipr1* re-expression was not found to affect the proliferation of 5TGM1 cells either in mono-culture (*P* = 0.985; Fig 3C) or in co-culture with the OP9 murine BM stromal cell line (*P* = 0.473; Fig 3D) over three days. Furthermore, the number of colonies formed by 5TGM1-Glipr1 cells in semi-solid medium did not differ from that of 5TGM1-EV control cells after 12 days (*P* = 0.264; Fig 3E).

### Re-expression of Glipr1 in 5TGM1 cells does not affect tumour growth *in vivo*

**Fig 4:**
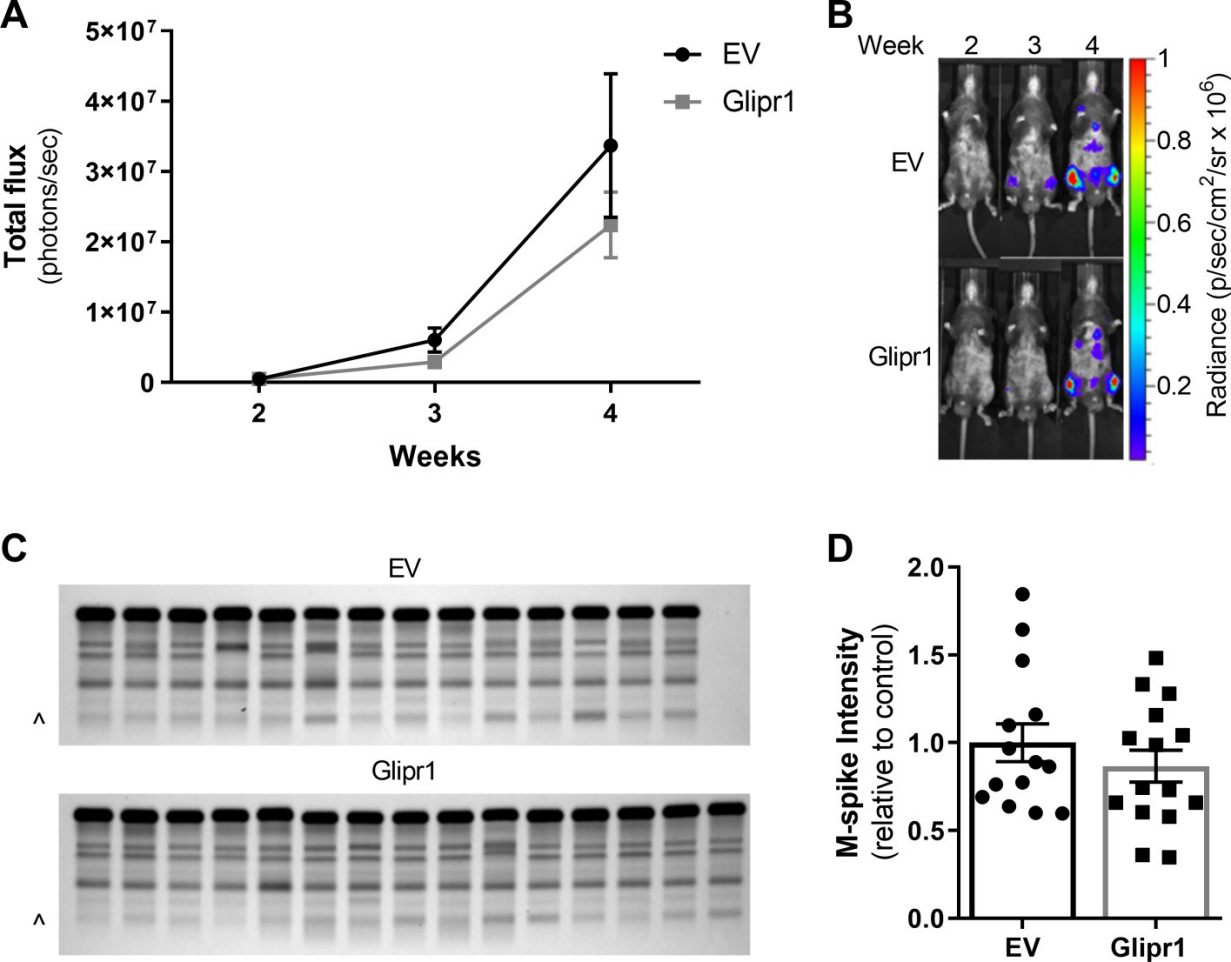
*Glipr1* overexpression in 5TGM1 cells does not affect tumour growth *in vivo*. KaLwRij mice were injected intravenously with 5 x 10^5^ 5TGM1-Glipr1 or 5TGM1-EV control cells. (**A**) Tumour burden in the mice was measured weekly from week 2 post-tumour cell inoculation by bioluminescence imaging and the signal from the ventral and dorsal scans were summed for each mouse. A graph of the total flux for the mice injected with 5TGM1-Glipr1 or 5TGM1-EV cells (left) and representative ventral scans of one mouse per cell line over time (right) are shown. (**B**) Serum was collected from the mice after four weeks and the M-spikes were measured by SPEP. M-spikes (^) on the SPEP gel (left) and the quantitated M-spike intensity (right), normalised to albumin and expressed relative to the EV control, are shown. Graphs depict the mean ± SEM of n = 14–15 mice per cell line from three independent experiments.

To determine the effect of Glipr1 on MM tumour growth *in vivo*, the 5TGM1-Glipr1 and 5TGM1-EV cell lines were injected intravenously into KaLwRij mice and tumour burden was monitored at weekly intervals by bioluminescence imaging. There was a trend towards reduced tumour burden at 4 weeks in the KaLwRij mice inoculated with 5TGM1-Glipr1 cells compared with those mice inoculated with 5TGM1-EV cells, but this decrease did not reach statistical significance (*P* = 0.246; Fig 4A). Tumour burden was also independently assessed at 4 weeks by measuring monoclonal paraprotein (M-spike) levels using serum protein electrophoresis (SPEP). The M-spike intensity showed the same trend toward reduced tumour burden in the 5TGM1-Glipr1 compared to the 5TGM1-EV group, but statistical significance was not reached (*P* = 0.451; Fig 4B).

### Generation of *Glipr1* knockout mice using CRIPSR-Cas9 genome editing

**Fig 5:**
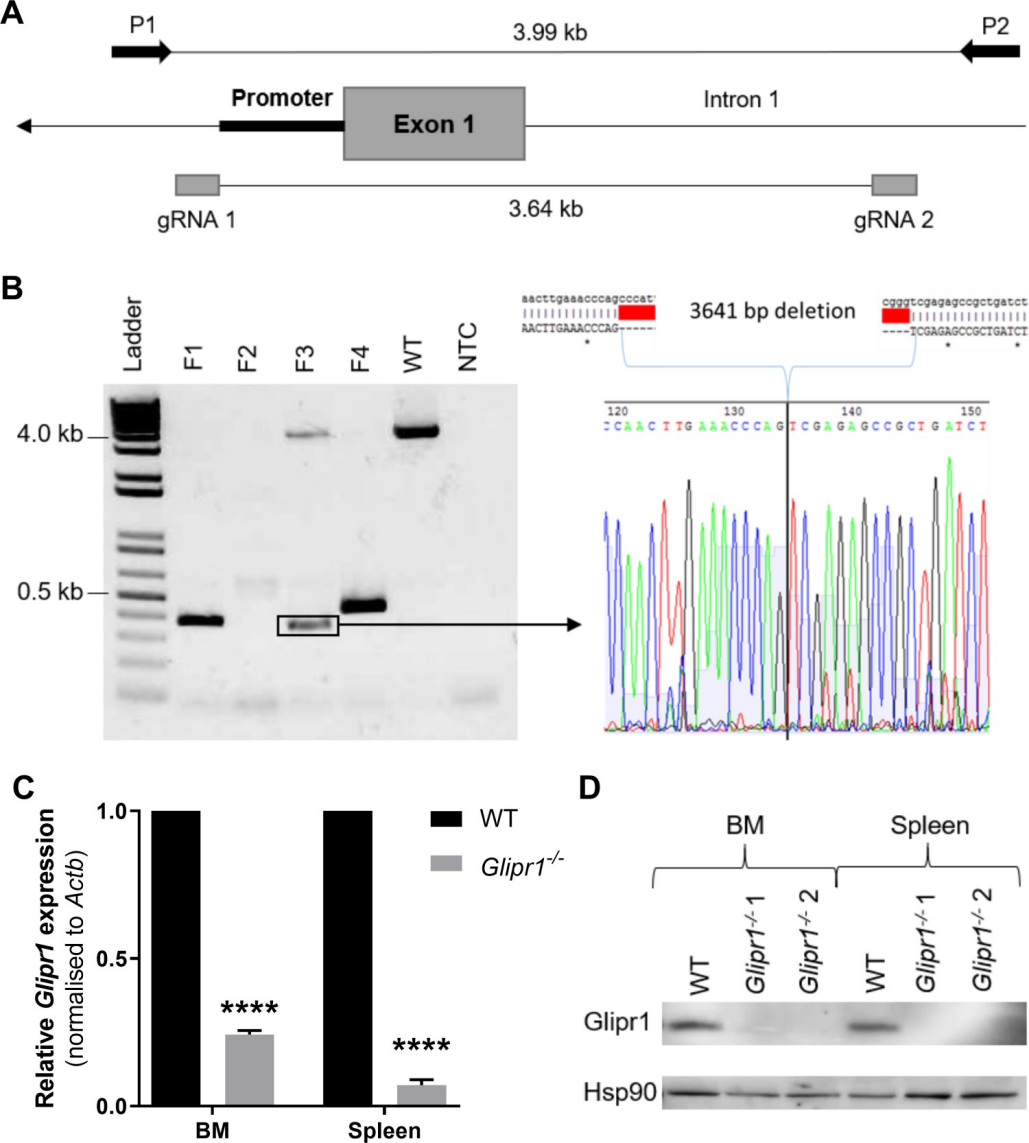
Generating *Glipr1* knockout mice (*Glipr1*^*-/-*^) using CRISPR-Cas9 genome editing. **(A**) Schematic showing the location of the gRNAs used for CRISPR-Cas9-mediated deletion of *Glipr1* exon 1 and the PCR primers (P1 & P2) used to screen founder mice for deletions. The direction of gene transcription is indicated by the arrow. (**B**) DNA samples from the four founder mice (F1-4) were screened for deletions of *Glipr1* exon 1 by PCR using primers P1 and P2 and the products were run on a 1% agarose gel (left). Sanger sequencing of the highlighted deletion band in F3 showed a 3,641 bp deletion between the two gRNA sites, which removed *Glipr1* exon 1 (right). NTC = no template control, WT = wildtype C57BL/6 mouse. (**C**) RT-qPCR for *Glipr1* mRNA was performed on RNA from the BM and spleen of *Glipr1*^*-/-*^ mice and WT control mice using primers in exons 3 and 4. *Glipr1* expression levels were normalised to *Actb* and were expressed relative to WT mice. Graph depicts the mean + SD of n = 2 mice per genotype. ****P < 0.0001, unpaired t test. (**D**) The levels of Glipr1 protein in the BM and spleen of *Glipr1*^*-/-*^ and WT mice was assessed by Western blot. Hsp90 was used as the loading control.

As *Glipr1* overexpression in MM PCs caused a trend towards reduced tumour growth in the aggressive KaLwRij-5TGM1 model, we hypothesised that the loss of *Glipr1* expression in mice may promote the development of premalignant and/or malignant PC expansions. To test this, Glipr1 knockout mice were generated using CRISPR-Cas9 genome editing technology. The strategy was to delete the first exon of the *Glipr1* gene using two guide RNAs (gRNAs), one upstream of the conserved promoter region and the other in the first intron of *Glipr1* (Fig 5A). C57BL/6 WT mouse zygotes were injected with Cas9 mRNA and both gRNAs, and were then transferred to pseudopregnant recipients, which resulted in the birth of four founder mice (F1-4). PCR genotyping coupled with Sanger sequencing revealed that three of the founders had at least one *Glipr1* allele in which the first exon was successfully deleted (Fig 5B). The *Glipr1* deletion allele of founder 3, a ~3.6 kb deletion encompassing exon 1 (Fig 5B), was selected for breeding to homozygosity because it did not involve any random insertions/deletions and belonged to the only male founder. This *Glipr1* deletion allele was backcrossed onto a C57BL/6 background for one generation and then bred to homozygosity to generate C57BL/*Glipr1*^*-/-*^ (*Glipr1*^*-/-*^) mice. To confirm successful gene knockout, RT-qPCR for *Glipr1* mRNA was performed on RNA from the B cell-rich BM and spleen of *Glipr1*^*-/-*^ mice and WT control mice. Using PCR primers in *Glipr1* exons 3 and 4, the expression of *Glipr1* mRNA transcripts in the BM and spleen of *Glipr1*^*-/-*^ mice was significantly reduced compared to that of WT mice (Fig 5C). While Glipr1 protein expression was observed in lysates from BM and spleen cells of WT mice, there was no Glipr1 protein detected in the BM or spleen of the *Glipr1*^*-/-*^ mice by Western blot (Fig 5D), confirming Glipr1 knockout in these mice.

### Analysis of B cell development in 12-week-old *Glipr1* knockout mice

**Fig 6:**
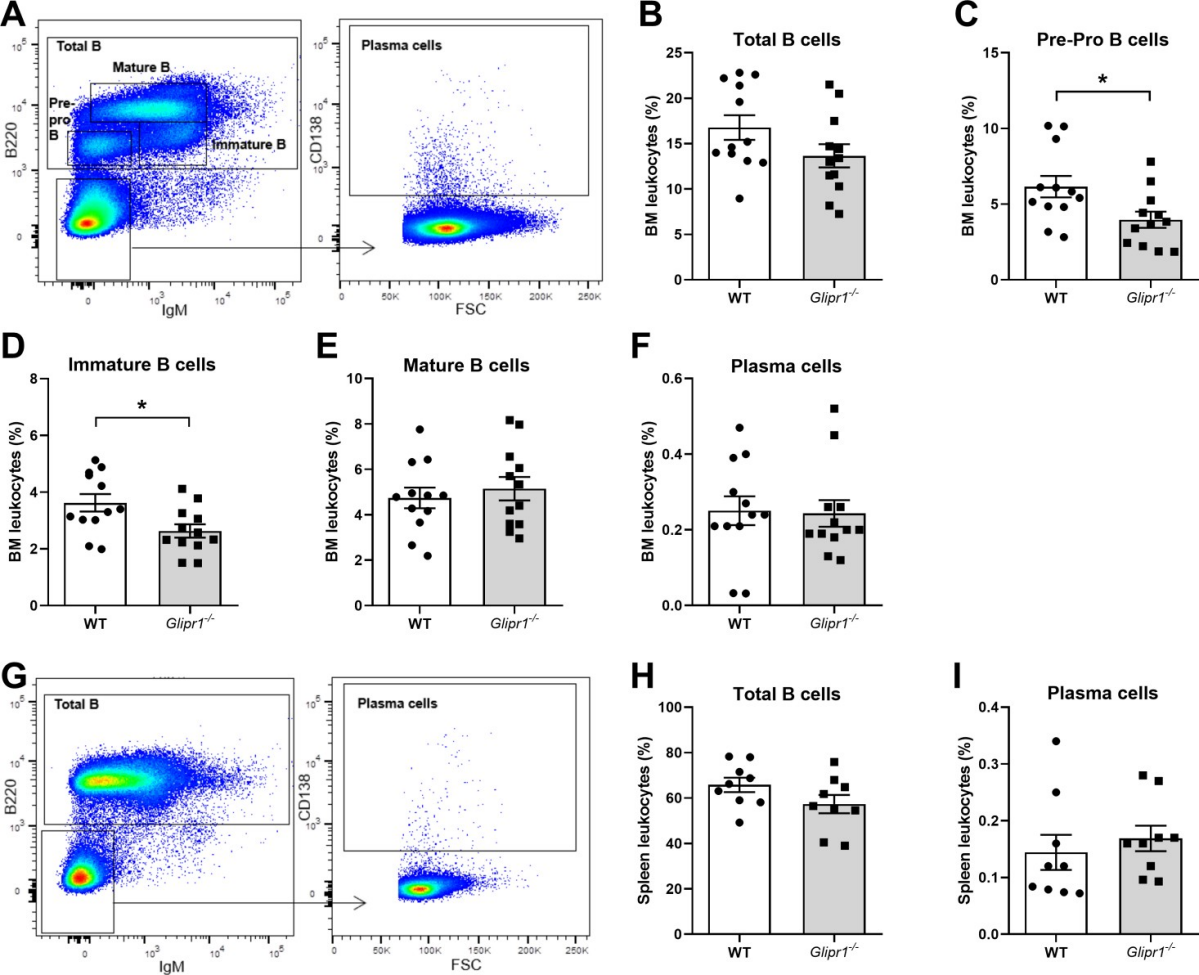
FACS analysis of B cell development in 12-week-old WT and *Glipr1*^*-/-*^ mice. Single cell suspensions from the BM and spleen were obtained from 12-week-old *Glipr1*^-/-^ mice and WT control mice. The cells were stained with anti-B220, anti-IgM and anti-CD138 antibodies and analysed by flow cytometry. BM cells were gated, as represented in (**A**), to show the percentage of total B cells (B220^+^; **B**), pre-pro B cells (B220^low^IgM^-^; **C**), immature B cells (B220^low^IgM^+^; **D**), mature B cells (B220^high^IgM^low^; **E**) and PCs (B220^-^IgM^-^CD138^+^; **F**) among total leukocytes. Spleen cells were gated, as represented in (**G**), to show the percentage of total B cells (**H**) and PCs (**I**) among total leukocytes. Graphs depict the mean ± SEM of n = 12 (**B**-**F**) or n = 9 (**H**&**I**) mice per genotype. *P < 0.05, Mann-Whitney U test.

*Glipr1* is reported to be expressed in murine B cells and PCs [53], therefore, we aimed to elucidate the effect of *Glipr1* knockout on normal haematopoiesis, particularly B cell development, in adult mice. Peripheral blood from 12-week-old *Glipr1*^*-/-*^ mice and WT mice was assessed using a HEMAVET analyser. No significant differences in the numbers of white or red blood cells, haemoglobin concentration, or other measured parameters were observed between the *Glipr1*^*-/-*^ mice and WT mice (S1 Table). In addition, BM was collected from 12-week-old *Glipr1*^*-/-*^ mice and WT mice and flow cytometric analyses of B cell populations were performed (Fig 6A). Although there was no difference in total B cells (*P* = 0.101; Fig 6B), a significant reduction in the percentage of pre-pro B cells (*P* = 0.024; Fig 6C) and immature B cells (*P* = 0.033; Fig 6D) was observed in the *Glipr1*^*-/-*^ mice compared with WT controls. No differences in the populations of mature B cells (*P* = 0.702; Fig 6E) or PCs (*P* = 0.369; Fig 6F) were observed. B cell populations in the spleens of the mice were also analysed by flow cytometry (Fig 6G) and no difference in total B cells *P* = 0.114; Fig 6H) and PCs (*P* = 0.181; Fig 6I) were observed between the *Glipr1*^*-/-*^ mice and WT controls. Furthermore, to assess whether *Glipr1* loss may predispose primary murine B cells to expansion, the proliferation of purified splenic B cells in response to *ex vivo* stimulation with IL-4 and LPS was measured. There was a trend towards increased proliferation of B cells from *Glipr1*^*-/-*^ mice compared to WT mice over three days, but statistical significance was not reached (*P* = 0.232; S3 Fig).

### Loss of Glipr1 does not affect B cell/PC expansions in 12-month-old mice

Previous studies have shown that *Glipr1*^*-/-*^ mice develop MM-like disease with late onset and incomplete penetrance [29]. Therefore, we hypothesised that the loss of Glipr1 would result in an increased and/or accelerated incidence of abnormal PC expansions in ageing mice. To test this, *Glipr1*^*-/-*^ mice and WT control mice were aged for 12 months and subjected to HEMAVET, flow cytometry and SPEP analyses. To investigate the possible effects of *Glipr1* loss on non-B cell populations that may indirectly impact on the PC population within the BM microenvironment, flow cytometric analyses were performed. No significant differences in the percentages of haematopoietic stem cells (HSCs; S4 Fig), monocytes, macrophages, granulocytes (S5 Fig) or endothelial cells (S6 Fig) in the BM, or mesenchymal stem cells (MSCs; S7 Fig) in the bone, were observed between the *Glipr1*^*-/-*^ and WT control mice.

**Fig 7:**
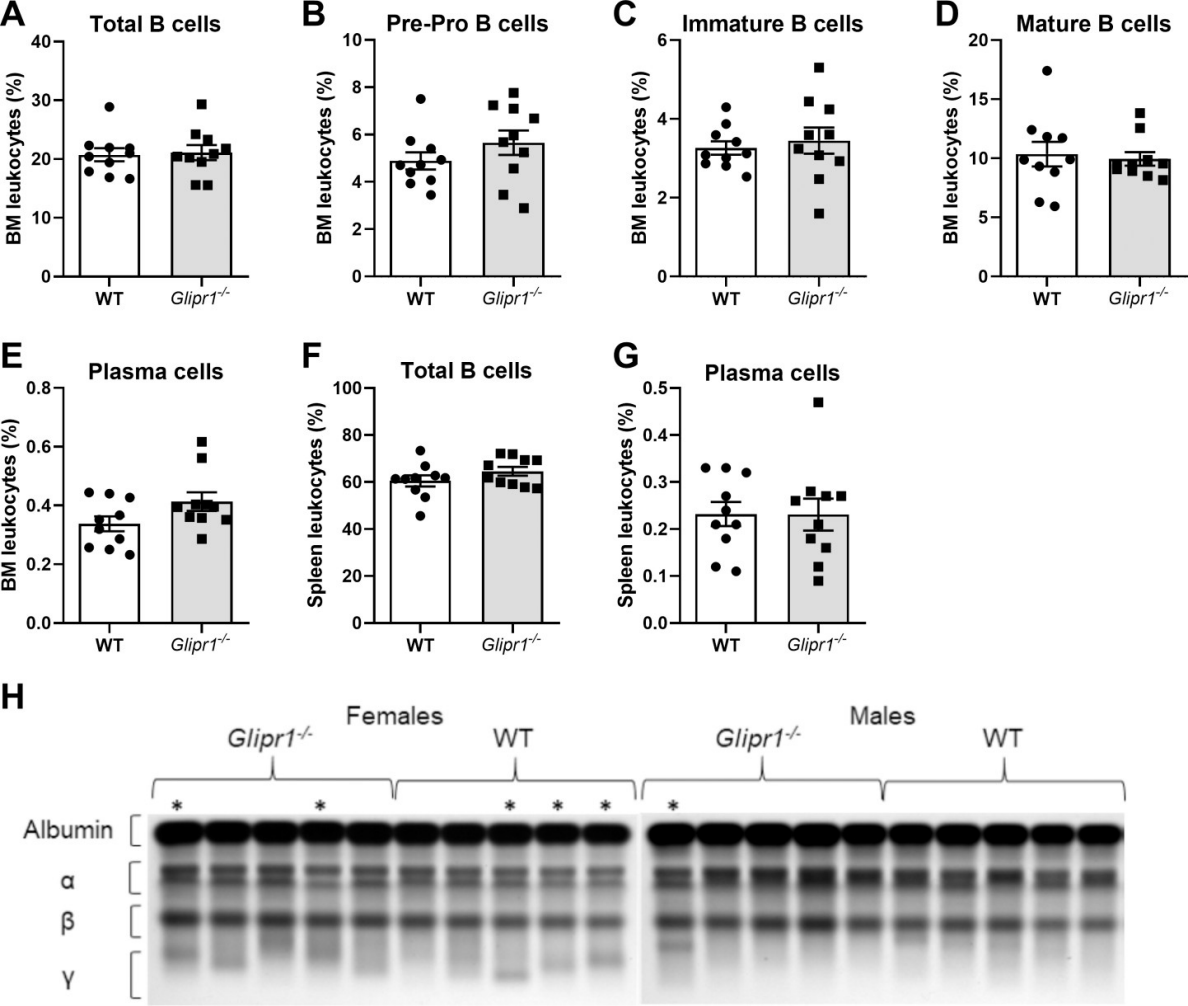
*Glipr1* knockout does not affect B cell or PC populations in 12-month-old mice. BM and spleen cells were prepared from 12-month-old *Glipr1*^*-/-*^ and WT control mice. Resultant single cell suspensions were stained with anti-B220, anti-IgM and anti-CD138 antibodies and analysed by flow cytometry. BM cells were gated to show the percentage of total B cells (B220^+^; **A**), pre-pro B cells (B220^low^IgM^-^; **B**), immature B cells (B220^low^IgM^+^; **C**), mature B cells (B220^high^IgM^low^; **D**) and PCs (B220^-^IgM^-^CD138^+^; **E**) among total leukocytes. Spleen cells were gated to show the percentage of total B cells (**F**) and PCs (**G**) among total leukocytes. Graphs depict the mean ± SEM of n = 10 mice per genotype. (**H**) Serum was collected by tail bleed from 12-month-old *Glipr1*^*-/-*^ mice and WT control mice and the presence of M-spikes was determined using SPEP (n = 10 mice per genotype). The SPEP gels are shown with the position of the albumin and globulin components of the serum indicated by brackets. * = mice with an M-spike by densitometry.

Analysis of peripheral blood parameters identified a significant reduction in the number of monocytes in *Glipr1*^*-/-*^ mice compared with WT controls (*P* = 0.015; S2 Table). No differences were observed in the other blood parameters measured (S2 Table). Flow cytometric analyses of B cell populations within the BM revealed no differences in the total B cell (*P* = 0.912; Fig 7A), pre-pro B cell (*P* = 0.248; Fig 7B), immature B cell (*P* = 0.631; Fig 7C), mature B cell (*P* = 0.684; Fig 7D) and PC (*P* = 0.171; Fig 7E) populations between *Glipr1*^*-/-*^ and WT control mice. Similarly, no differences in the total B cell (*P* = 0.306; Fig 7E) and PC (*P* = 0.781; Fig 7F) populations in the spleen were observed. The development of clonal PC expansions was assessed by looking for M-spikes in the serum of the 12-month-old *Glipr1*^*-/-*^ mice and WT mice using SPEP. In total, five out of ten female mice showed evidence of an M-spike compared to only one out of ten male mice (Fig 6H), which was consistent with a previous report of higher M-spike incidence in one-year-old female C57BL mice [54]. Three out of ten *Glipr1*^*-/-*^ mice were found to have an M-spike, which was equal to the incidence of M-spikes amongst the WT mice (Fig 6H). Together, these results suggest that the loss of Glipr1 does not promote the development of PC expansions in C57BL mice up to one year of age.

## Discussion

The genetics of MM PCs are extremely complex with the majority of MM patients exhibiting a subclonal disease structure with a high degree of genetic heterogeneity and a distinct lack of recurrent mutations [8, 10, 11]. While an array of chromosomal rearrangements, nucleotide variations, transcriptional alterations and epigenetic changes are known to occur within PCs during disease pathogenesis, the complete complement of genetic abnormalities that contribute to driving MM development remain to be fully elucidated [55–57]. Here, we show that *GLIPR1* expression is significantly reduced in PCs from MM patients compared to normal controls, with the majority of MM patients having *GLIPR1* mRNA levels below the normal range. This is consistent with the previously reported down-regulation of *GLIPR1* expression in the PCs of light-chain amyloidosis patients compared with normal controls [40], suggesting that this may be a common genetic event in PC malignancies. The median *GLIPR1* expression levels in the PCs of MGUS patients were most similar to those levels in normal controls in one microarray dataset and, conversely, to those levels in MM patients in the second dataset. Hence, the stage at which *GLIPR1* expression is down-regulated during MM disease development remains unclear. In addition, we found that *GLIPR1* expression was reduced in a subset of HMCLs, which is consistent with the previous finding that the HMCLs U266 and RPMI-8226 have significantly reduced *GLIPR1* expression compared to normal B cells [29]. Together, these data suggest a role for the down-regulation of *GLIPR1* in promoting MM disease development.

In this study, *GLIPR1* expression was found to be lower in MM patients belonging to the hyperdiploid (HY) and low bone disease (LB) UAMS molecular subgroups compared to the MAF, MMSET and Cyclin translocation subgroups, although the mechanisms underlying these associations remain unclear. As the HY and LB subgroups have previously been found to have a more favourable prognosis [43], the enrichment of MM patients with reduced *GLIPR1* expression in these subgroups is consistent with our finding that *GLIPR1* expression does not impact on overall survival. In the context of murine MM, the expression of *Glipr1* was found to be absent in the KaLwRij tumour-derived 5TGM1 MM cell line compared to detectable levels in PCs from healthy KaLwRij and WT mice, suggesting that *Glipr1* down-regulation may also play a role in the development of murine MM. To investigate whether loss of *Glipr1* expression is a common occurrence in the spontaneous MM tumours that arise in KaLwRij mice, other MM cells derived from different KaLwRij donors, such as the 5T2 cell line [50, 58–60], could be analysed.

The mechanism(s) by which *Glipr1*/*GLIPR1* expression is down-regulated in the context of human and murine MM remains unknown. Hemizygous chromosomal deletions encompassing *GLIPR1* have been reported in 9.4% of MM patients [39], suggesting that this may be a mechanism that contributes to the down-regulation of *GLIPR1* in human PCs. The potential role of chromosomal deletion in reducing *GLIPR1* expression in PCs could be examined by correlating the *GLIPR1* copy number (as determined by fluorescence in situ hybridization) and *GLIPR1* expression levels in a panel of HMCLs. Analysis of copy number aberrations in the 5TGM1 cell line compared to the KaLwRij genome did not reveal any deletions encompassing *Glipr1* [61], suggesting that the loss of *Glipr1* expression occurs by an alternative mechanism in these murine MM cells. Reduced *GLIPR1* expression in prostate cancer cells was shown to be primarily caused by aberrant DNA hypermethylation [26]. Given that the down-regulation of tumour suppressor genes by hypermethylation of promoter CpG sites is a common feature of MM PCs [56, 62], it is possible that this mechanism underlies reduced *GLIPR1* expression in at least some patients. This could be examined by correlating differential methylation of specific CpGs within the *GLIPR1* promoter with gene expression levels in a panel of HMCLs.

Overexpression of GLIPR1 in prostate, bladder and lung cancer, as well as osteosarcoma cells, has been demonstrated to reduce tumour cell proliferation and/or colony formation *in vitro* [24, 25, 27, 29]. In contrast, neither the overexpression of GLIPR1 in the H929 HMCL nor the re-expression of Glipr1 in the 5TGM1 murine MM cell line affected basal tumour cell proliferation or colony formation *in vitro*. The interaction between malignant PCs and accessory cells of the BM microenvironment, including immune cells [63], stromal cells [64] and endothelial cells [65], is critical for MM disease manifestation. Immune system avoidance [66] and new blood vessel formation [67] are key aspects of MM disease development that can only be modelled in an immune competent animal model. Given the integral role of the BM microenvironment on MM tumour development and the inability to fully recapitulate its complexities *in vitro* [68], the 5TGM1/KaLwRij murine model of MM was used to assess the effect of Glipr1 re-expression on MM tumour growth *in vivo*. A reduction in tumour burden was observed in mice inoculated with 5TGM1-Glipr1 cells compared to mice inoculated with 5TGM1-EV cells after four weeks, but this difference did not reach statistical significance in the 5TGM1/KaLwRij model. This result contrasts with the previously reported significant reduction in tumour burden following intra-tumoral administration of an adenoviral *Glipr1* expression vector in an orthotopic, metastatic mouse model of prostate cancer [30]. Given that the highly progressive nature of the KaLwRij-5TGM1 model of MM [69], it was our contention that these findings did not exclude the possibility that reduced *GLIPR1* expression may contribute to the development of PC expansions/malignancy. Our preclinical model was powered at 80% to detect a 50% reduction in tumour volume at the experimental endpoint.

To examine this, we generated *Glipr1* knockout (*Glipr1*^-/-^) mice by removing the first exon of the gene using CRISPR-Cas9 genome editing. As previous RNA-seq data has shown that *Glipr1* is expressed throughout B cell lineage development, with the highest expression levels observed in PCs [53], we hypothesised that Glipr1 knockout may disrupt B cell development in mice. A significant reduction in the percentages of pre-pro and immature B cells, but not mature B cells or PCs, was observed in the BM of *Glipr1*^-/-^ mice compared with WT controls at 12 weeks of age. However, this decrease in early B cell populations was not replicated in 12-month-old mice. Hence, further research is required to determine whether GLIPR1 plays a role in early B cell development. It remains a possibility that Glipr1 is necessary for optimal early B cell survival and/or retention within the BM in young adult mice, and that mature B cells are less dependent on Glipr1 for their survival/retention. Whether this effect is driven by a B cell intrinsic lack of Glipr1, or by a compromised B cell developmental niche caused by Glipr1-null BM accessory cells remains undetermined. The finding that red and white blood cell numbers were similar between *Glipr1*^-/-^ and WT control mice at 12 weeks of age suggests that Glipr1 is unlikely to have a major role in haematopoiesis. However, we found that resting splenic B cells from *Glipr1*^-/-^ mice exhibited a trend towards increased proliferation in response to stimulation with LPS and IL-4, suggesting that Glipr1 may have a role in regulating B cell function, specifically inhibiting B cell expansion. This result is consistent with the possibility that reduced *Glipr1* expression may promote PC expansions *in vivo*.

In this study, the effect of Glipr1 loss on the development of clonal PC expansions was assessed in 12-month-old *Glipr1*^-/-^ and WT control mice. No differences in PC populations were observed by flow cytometric analyses of BM and spleen cells from *Glipr1*^-/-^ mice compared with WT control mice. In addition, SPEP analysis showed that the incidence of M-spikes, which is evidence of a possible clonal PC expansion, was 30% for both the *Glipr1*^-/-^ and WT mice. This is in agreement with the previously reported M-spike frequency of ~25–30% in one-year-old C57BL mice [36, 54]. Together, these data suggest that the loss of Glipr1 does not promote the development of PC proliferative disorders in mice up to one year of age. These findings contrast with the previously reported propensity of *Glipr1*^-/-^ mice to develop plasmacytomas [29]. However, the low penetrance (~17%) and late onset (no mortality until at least 15 months of age) of tumours in these mice suggests that the cohort size and length of monitoring of the *Glipr1*^-/-^ mice in this study may have been insufficient to observe potentially enhanced PC tumorigenesis. In addition, the previously described *Glipr1* knockout mice had a C57BL6/129Sv (1:1) hybrid genetic background [29] and studies have shown that tumour penetrance and onset are increased in transgenic mice with a 129Sv background compared to a C57BL/6 background [70–73]. Hence, it is likely that the previously described *Glipr1* knockout mice were more susceptible to developing PC abnormalities compared to those generated in this study, which were on a pure C57BL/6 background. Therefore, in order to further assess the impact of Glipr1 loss on the development of PC malignancies *in vivo*, future studies should include ageing a larger cohort of our *Glipr1*^-/-^ mice over a longer timeframe (~ 2 years).

Overexpression of *MYC* due to chromosomal rearrangements and/or other genetic abnormalities is a common feature of MM and has been shown to be a potent driver of the progression from MGUS to MM [33–36]. Here, *in silico* analysis showed that *GLIPR1* expression is inversely correlated with *MYC* expression in the PCs of MM patients, suggesting that *GLIPR1* may contribute to the negative regulation of oncogenic *MYC* expression. This finding is consistent with the previous report of *GLIPR1* expression being inversely correlated with *MYC* expression in prostate cancer patient samples [32]. The same study also found that *GLIPR1* overexpression in prostate cancer cell lines led to a reduction in *MYC* expression due to GLIPR1 directly decreasing *MYC* transcription as well as promoting the ubiquitination and degradation of MYC protein [32]. In contrast, the re-expression of Glipr1 in 5TGM1 cells did not alter *Myc* mRNA levels, suggesting that Glipr1 does not regulate *Myc* expression in these cells. Hence, it remains unclear whether GLIPR1 directly regulates *MYC* expression in PCs. It was previously shown that *MYC* overexpression and loss of *Glipr1* expression co-operate to promote prostate cancer development *in vivo* [32]. Hence, their potential co-operation in promoting the malignant transformation of PCs could be tested by generating *Glipr1*^-/-^ mice that also have PC-specific *Myc* overexpression (e.g., using the Vk*MYC transgene [36]) and then monitoring them for clonal PC expansions/malignancies,.

In conclusion, this is the first study to demonstrate that *GLIPR1* expression is frequently reduced in the PCs of MM patients and that *Glipr1* expression is lost in the 5TGM1 murine MM cell line. Despite this, the overexpression of *GLIPR1*/*Glipr1* in a HMCL/5TGM1 cells did not significantly alter tumour cell growth *in vitro* or *in vivo*. In addition, *Glipr1* knockout mice did not show evidence of increased monoclonal PC expansions up to one year of age. Together these results suggest that GLIPR1 is unlikely to be a potent tumour suppressor in MM, but further work is required to determine whether its down-regulation may contribute to disease development.

## Supporting information

S1 Fig The expression of *GLIPR1* in MM PCs differs between molecular subgroups, does not affect overall survival and is inversely correlated with *MYC* expression. (**A**) MM patients from microarray dataset GSE4581 (n = 414) were stratified into molecular subgroups based on the UAMS criteria; namely, patients characterised by increased proliferation-related genes (PR), chromosomal translocations involving cyclin D1 and cyclin D3 (CD1 and CD2), MAF (MF) or MMSET (MS), as well as patients exhibiting hyperdiploidy (HY) and decreased prevalence of lytic bone disease (LB). The expression of *GLIPR1* was analysed in each subset. Box and whiskers plots show the median, interquartile range, and minimum and maximum values for each subset; ^#^*P* < 0.01 relative to MF and MS, ^*P* < 0.01 relative to CD2; Kruskal-Wallis test with Dunn’s multiple comparison tests. (**B**) Kaplan–Meier plots of overall survival are shown for newly diagnosed MM patients stratified on the basis of median CD138^+^ PC *GLIPR1* expression, derived from microarray dataset E-TABM-1138 (n = 142). (**C-E**) *GLIPR1* expression levels in the PCs of newly diagnosed MM patients from E-GEOD-6477 (n = 69; **C**), GSE4581 (n = 414; **D**) and E-GEOD-16122 (n = 133; **E**) were plotted against the expression levels of *MYC*. Pearson correlation r coefficient and *P* values are shown. (**F**) Kaplan–Meier plots of overall survival are shown for newly diagnosed MM patients stratified on the basis of median CD138^+^ PC *MYC* expression, derived from microarray dataset E-TABM-1138 (n = 142).
(TIF)

S2 Fig *Glipr1* overexpression does not affect *Myc* expression levels in 5TGM1 cells. RT-qPCR for *Myc* mRNA was performed on RNA from 5TGM1-EV cells and 5TGM1-GLIPR1 cells. *Myc* expression levels were normalised to *Actb* and were expressed relative to 5TGM1-EV cells. Graph depicts the mean + SD of triplicates. *P* = 0.799, unpaired t test.
(TIF)

S3 Fig No difference in *ex vivo* proliferation of primary B cells from *Glipr1*^-/-^ compared with WT control mice. Purified splenic B cells from 12-week-old WT and *Glipr1*^-/-^ mice were cultured in the presence of IL-4 and LPS. Cell proliferation was measured by a WST-1 assay three days after stimulation. Graph depicts the mean + SD of n = 3 independent experiments. *P* = 0.232, paired t test.
(TIF)

S4 Fig FACS analysis of HSCs in the BM of 12-month-old *Glipr1*^*-/-*^ mice. BM was collected from 12-month-old *Glipr1*^*-/-*^ and WT control mice and single cell suspensions were prepared. The cells were stained with lineage markers, anti-Sca1, anti-CD117, anti-CD135 and anti-CD34 antibodies and analysed by flow cytometry. (**A**) Representative flow plots showing the gating strategy used to define haematopoietic stem progenitor cells (HSPCs; Lin^-^Sca1^+^CD117^+^), short-term haematopoietic stem cells (ST-HSCs; Lin^-^Sca1^+^CD117^+^CD135^-^CD34^-^) and long-term haematopoietic stem cells (LT-HSCs; Lin^-^Sca1^+^CD117^+^CD135^-^CD34^+^). Graphs show the percentage of HSPCs among Lin^-^ cells (**B**), and ST-HSCs (**C**) and LT-HSCs (**D**) among total HSPCs. Graphs depict the mean ± SEM of n = 10 mice per genotype.
(TIF)

S5 Fig FACS analysis of monocytes/macrophages and granulocytes in the BM of 12-month-old *Glipr1*^*-/-*^ mice. BM was collected from 12-month-old *Glipr1*^*-/-*^ and WT control mice and single cell suspensions were prepared. The cells were stained with anti-CD11b, anti-F4/80, anti-CD169 and anti-Ly6G antibodies and analysed by flow cytometry. (**A**) Representative flow plots showing the gating strategy used to define monocytes (CD11b^+^F4/80^+^CD169^-^Ly6G^-^), macrophages (CD11b^+^F4/80^+^CD169^+^) and granulocytes (CD11b^+^F4/80^-^CD169^-^Ly6G^+^). Graphs show the percentage of monocytes (**B**), macrophages (**C**) and granulocytes (**D**) among total leukocytes. Graphs depict the mean ± SEM of n = 10 mice per genotype.
(TIF)

S6 Fig FACS analysis of endothelial cells in the BM of 12-month-old *Glipr1*^*-/-*^ mice. BM was collected from 12-month-old *Glipr1*^*-/-*^ and WT control mice and single cell suspensions were prepared. The cells were stained with lineage markers, anti-CD11b, anti-CD45, anti-CD31 and anti-CD144 antibodies and analysed by flow cytometry. (**A**) Representative flow plots showing the gating strategy used to define total endothelial cells (Lin^-^CD45^-^CD31^+^) and mature endothelial cells (Lin^-^CD45^-^CD31^+^CD144^+^). Graphs show the percentage of endothelial cells (**B**) and mature endothelial cells (**C**) among Lin^-^CD45^-^ BM cells. Graphs depict the mean ± SEM of n = 10 mice per genotype.
(TIF)

S7 Fig FACS analysis of mesenchymal stem cells in the compact bone of 12-month-old *Glipr1*^*-/-*^ mice. Compact bone (CB) was collected from 12-month-old *Glipr1*^*-/-*^ and WT control mice and single cell suspensions were prepared. The cells were stained with lineage markers, anti-CD45, anti-CD31, anti-CD51 and anti-Sca1 antibodies and analysed by flow cytometry. (**A**) Representative flow plots showing the gating strategy used to define mesenchymal stem cells (MSCs; Lin^-^CD45^-^CD31^-^CD51^-^Sca1^+^). (**B**) Graph shows the percentage of MSCs among Lin^-^CD45^-^CD31^-^ CB cells. Graph depicts the mean ± SEM of n = 10 mice per genotype.
(TIF)

S1 Table Haematological parameters in the peripheral blood of 12-week-old *Glipr1*^*-/-*^ mice. Peripheral blood was collected by a tail bleed from 12-week-old *Glipr1*^*-/-*^ mice and WT control mice and was assessed on a HEMAVET analyser (n = 7/genotype). Data are given as mean ± SD.
(XLSX)

S2 Table Haematological parameters in the peripheral blood of 12-month-old *Glipr1*^*-/-*^ mice. Peripheral blood was collected by a tail bleed from 12-month-old *Glipr1*^*-/-*^ mice and WT control mice and was assessed on a HEMAVET analyser (n = 10/genotype). Data are given as mean ± SD. *P < 0.05, **P < 0.01, Mann-Whitney U test.
(XLSX)

S1 File Original blot and gel images contained in the manuscript’s figures. (PDF)
