# Supplementary figures and images for: GLIPR1 expression is reduced in multiple myeloma but is not a tumour suppressor in mice
Source: PLoS One. 2020 Jan 29;15(1):e0228408. doi: 10.1371/journal.pone.0228408 (PMC6988976; doi:10.1371/journal.pone.0228408)

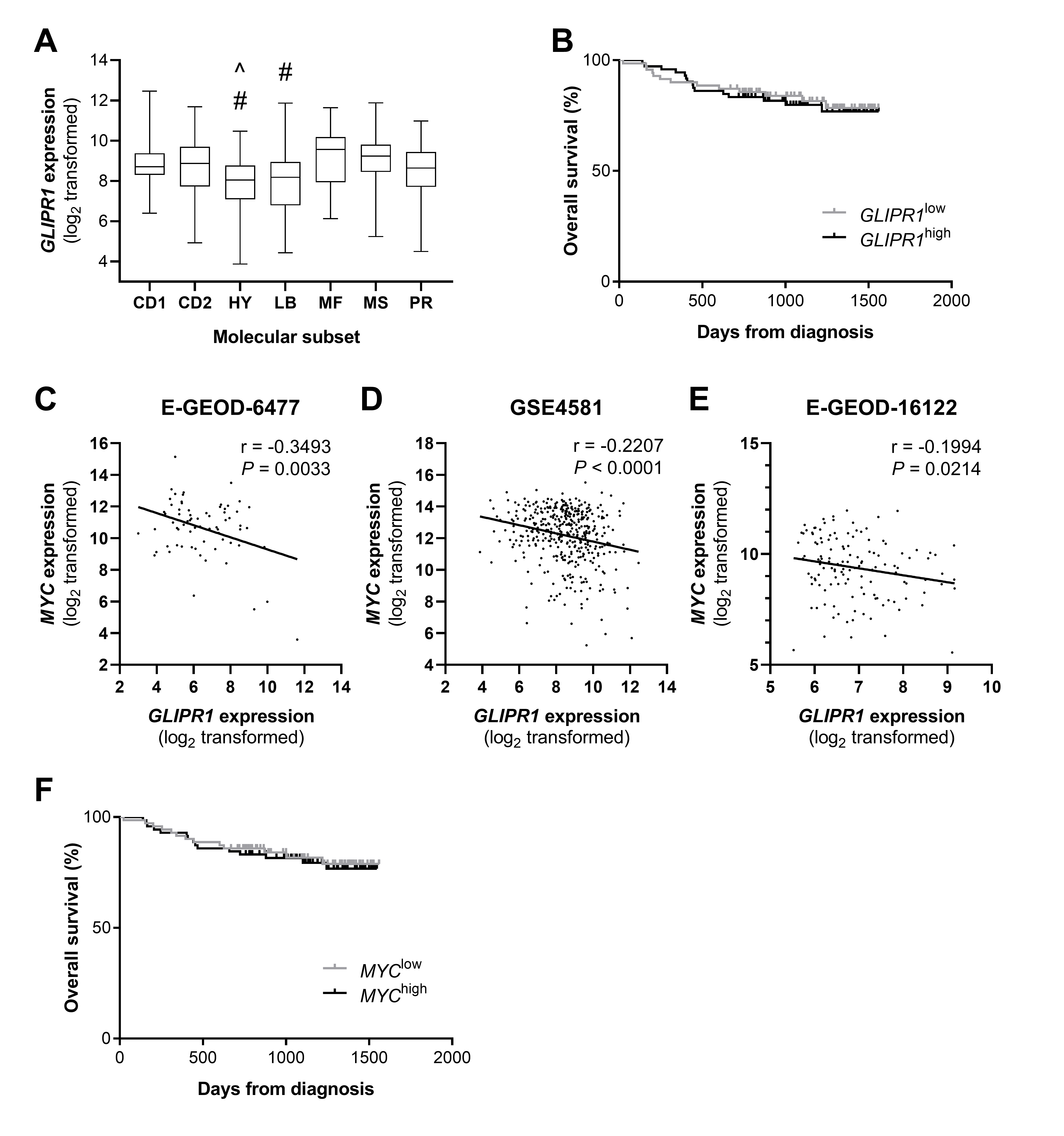

Supplement: S1 Fig — (A) MM patients from microarray dataset GSE4581 (n = 414) were stratified into molecular subgroups based on the UAMS criteria; namely, patients characterised by increased proliferation-related genes (PR), chromosomal translocations involving cyclin D1 and cyclin D3 (CD1 and CD2), MAF (MF) or MMSET (MS), as well as patients exhibiting hyperdiploidy (HY) and decreased prevalence of lytic bone disease (LB). The expression of GLIPR1 was analysed in each subset. Box and whiskers plots show the median, interquartile range, and minimum and maximum values for each subset; #P < 0.01 relative to MF and MS, ^P < 0.01 relative to CD2; Kruskal-Wallis test with Dunn’s multiple comparison tests. (B) Kaplan–Meier plots of overall survival are shown for newly diagnosed MM patients stratified on the basis of median CD138+ PC GLIPR1 expression, derived from microarray dataset E-TABM-1138 (n = 142). (C-E) GLIPR1 expression levels in the PCs of newly diagnosed MM patients from E-GEOD-6477 (n = 69; C), GSE4581 (n = 414; D) and E-GEOD-16122 (n = 133; E) were plotted against the expression levels of MYC. Pearson correlation r coefficient and P values are shown. (F) Kaplan–Meier plots of overall survival are shown for newly diagnosed MM patients stratified on the basis of median CD138+ PC MYC expression, derived from microarray dataset E-TABM-1138 (n = 142). (TIF) [file pone.0228408.s001.tif]

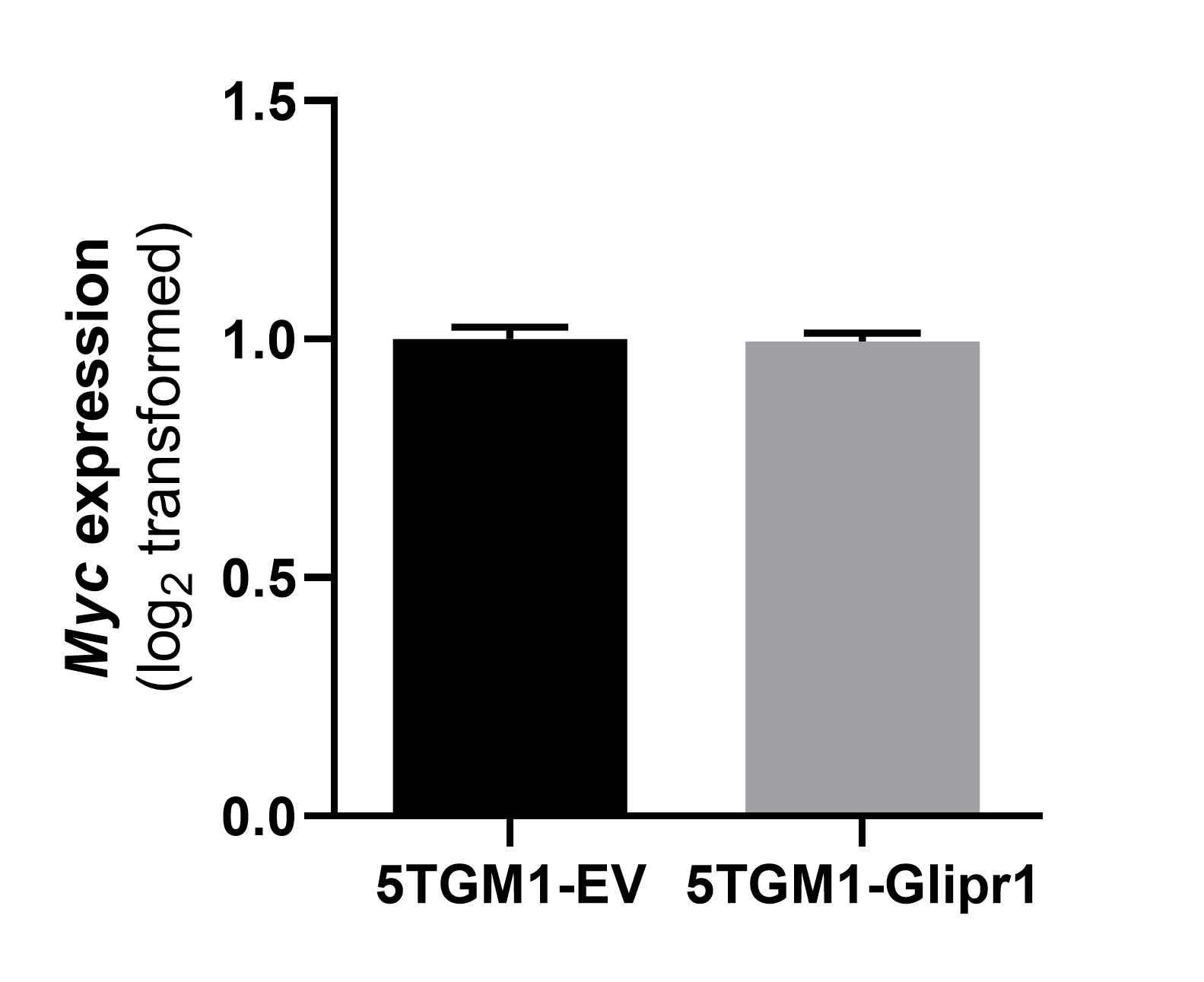

Supplement: S2 Fig — RT-qPCR for Myc mRNA was performed on RNA from 5TGM1-EV cells and 5TGM1-GLIPR1 cells. Myc expression levels were normalised to Actb and were expressed relative to 5TGM1-EV cells. Graph depicts the mean + SD of triplicates. P = 0.799, unpaired t test. (TIF) [file pone.0228408.s002.tif]

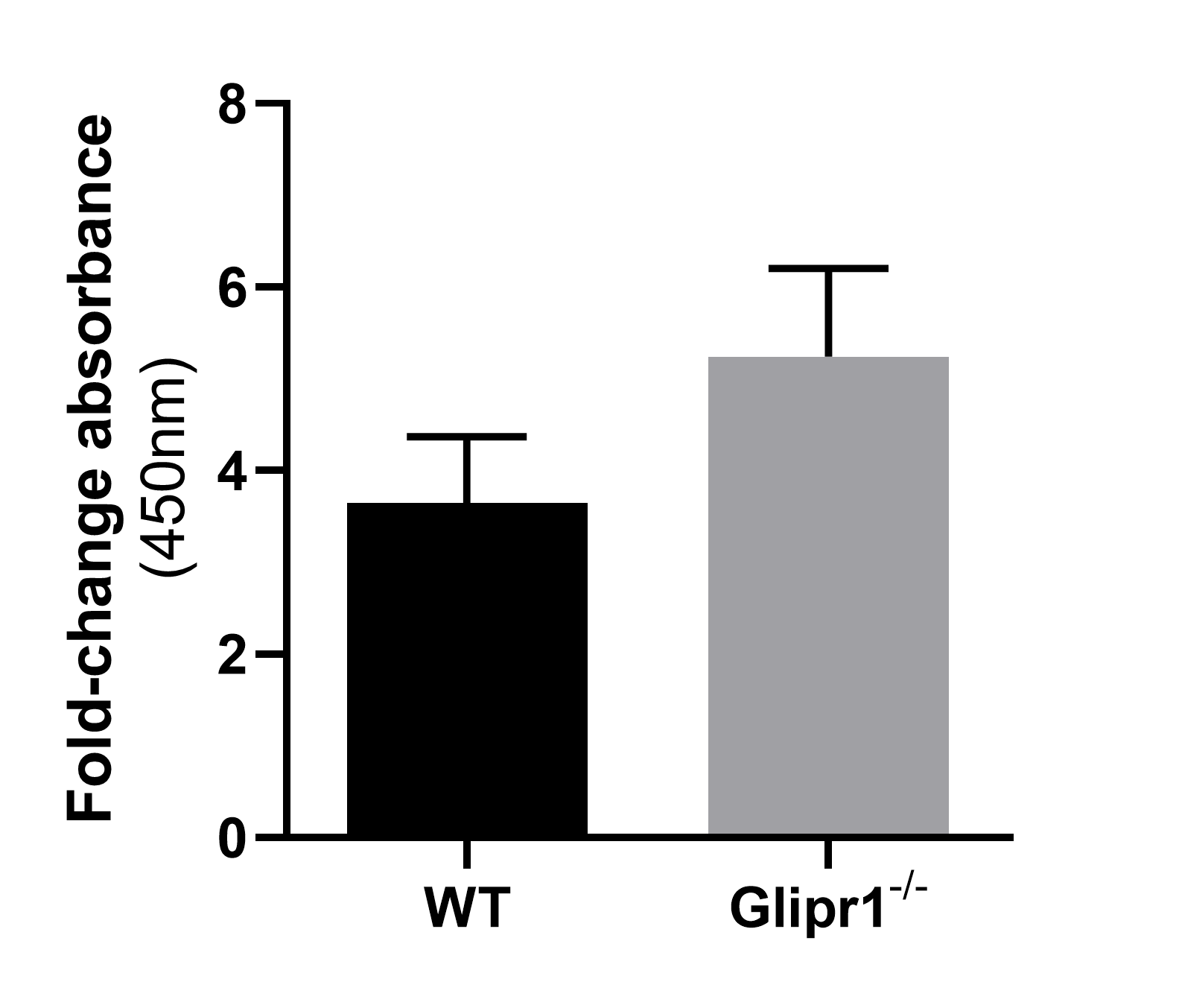

Supplement: S3 Fig — Purified splenic B cells from 12-week-old WT and Glipr1-/- mice were cultured in the presence of IL-4 and LPS. Cell proliferation was measured by a WST-1 assay three days after stimulation. Graph depicts the mean + SD of n = 3 independent experiments. P = 0.232, paired t test. (TIF) [file pone.0228408.s003.tif]

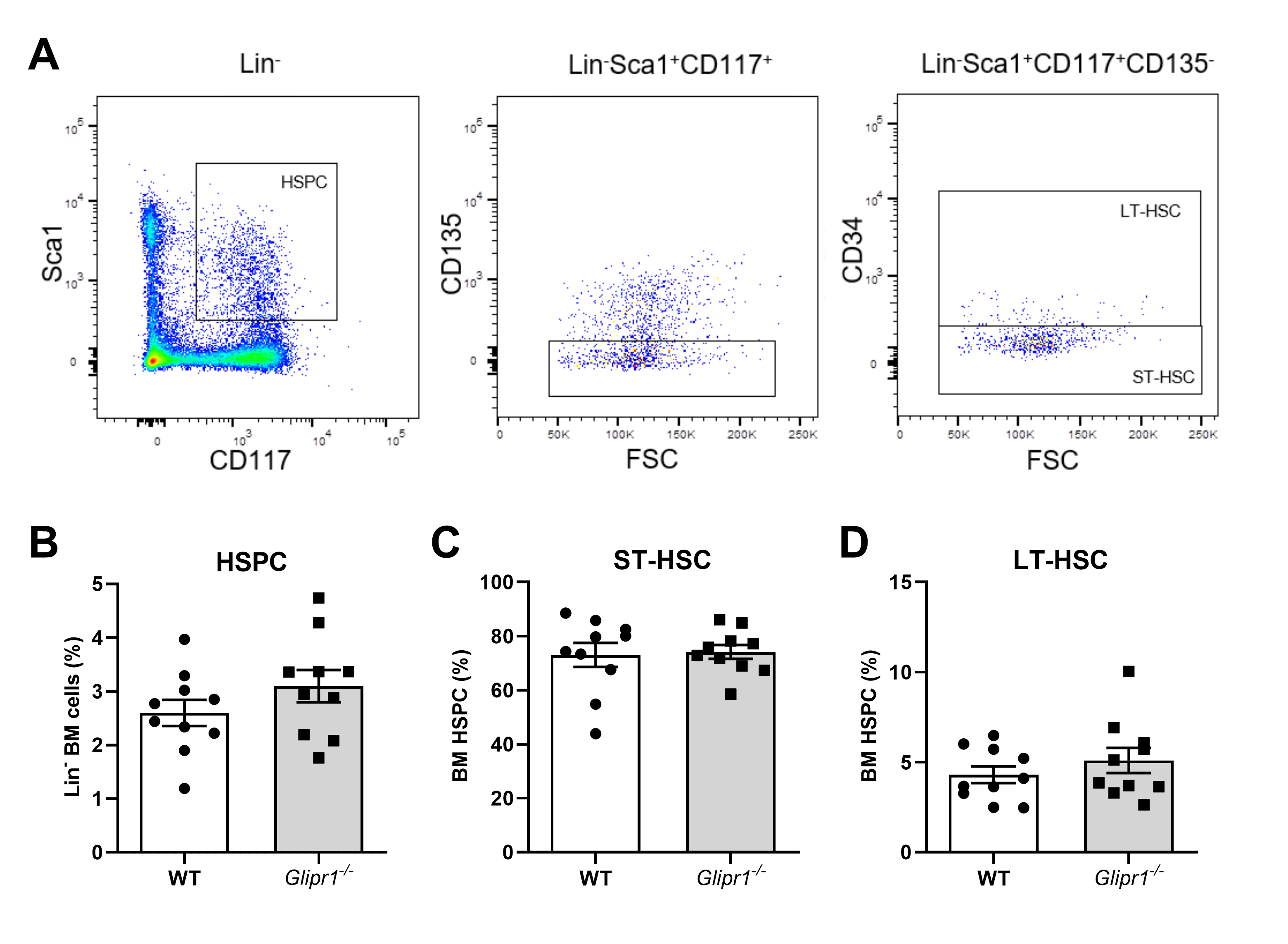

Supplement: S4 Fig — BM was collected from 12-month-old Glipr1-/- and WT control mice and single cell suspensions were prepared. The cells were stained with lineage markers, anti-Sca1, anti-CD117, anti-CD135 and anti-CD34 antibodies and analysed by flow cytometry. (A) Representative flow plots showing the gating strategy used to define haematopoietic stem progenitor cells (HSPCs; Lin-Sca1+CD117+), short-term haematopoietic stem cells (ST-HSCs; Lin-Sca1+CD117+CD135-CD34-) and long-term haematopoietic stem cells (LT-HSCs; Lin-Sca1+CD117+CD135-CD34+). Graphs show the percentage of HSPCs among Lin- cells (B), and ST-HSCs (C) and LT-HSCs (D) among total HSPCs. Graphs depict the mean ± SEM of n = 10 mice per genotype. (TIF) [file pone.0228408.s004.tif]

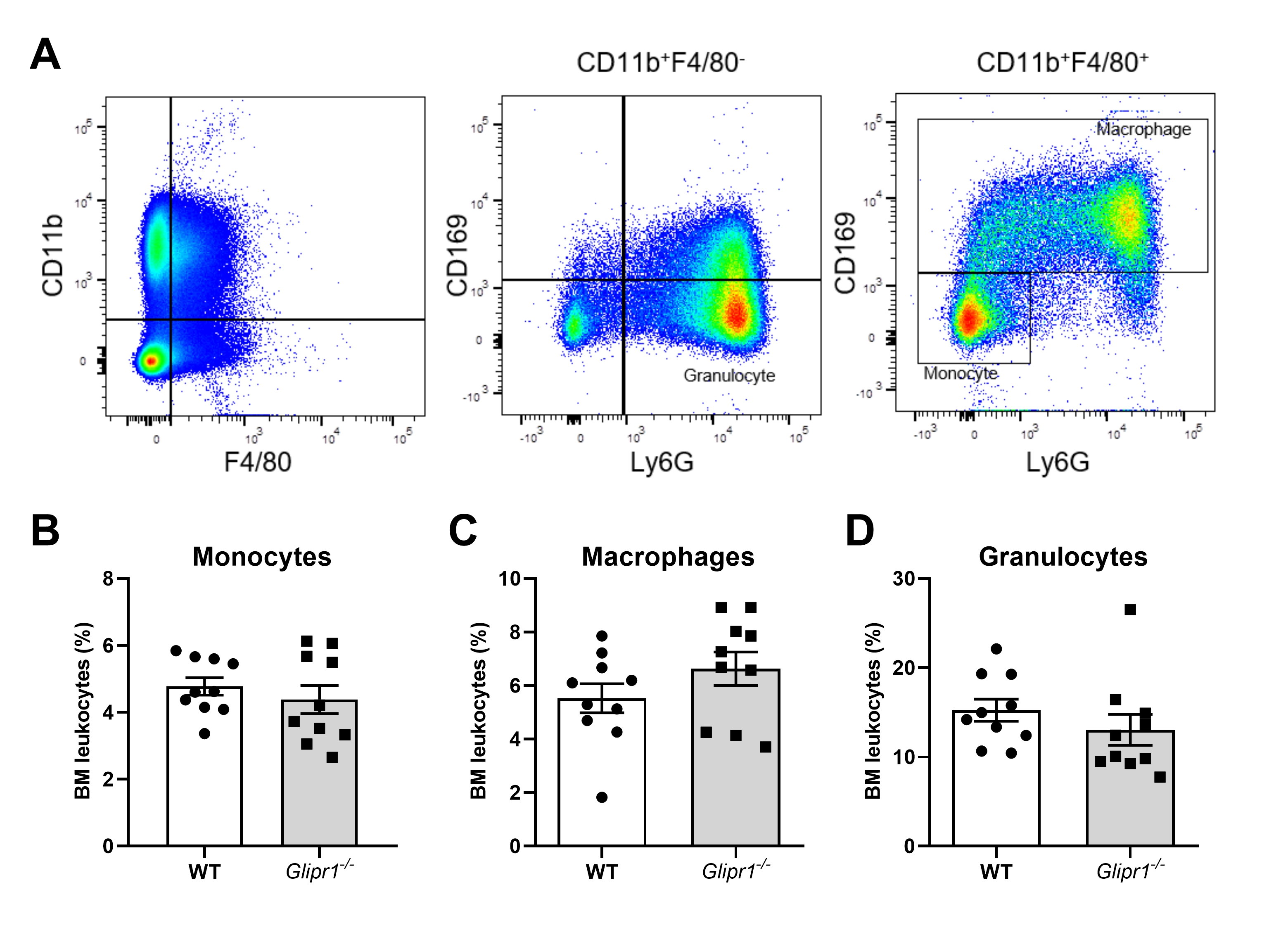

Supplement: S5 Fig — BM was collected from 12-month-old Glipr1-/- and WT control mice and single cell suspensions were prepared. The cells were stained with anti-CD11b, anti-F4/80, anti-CD169 and anti-Ly6G antibodies and analysed by flow cytometry. (A) Representative flow plots showing the gating strategy used to define monocytes (CD11b+F4/80+CD169-Ly6G-), macrophages (CD11b+F4/80+CD169+) and granulocytes (CD11b+F4/80-CD169-Ly6G+). Graphs show the percentage of monocytes (B), macrophages (C) and granulocytes (D) among total leukocytes. Graphs depict the mean ± SEM of n = 10 mice per genotype. (TIF) [file pone.0228408.s005.tif]

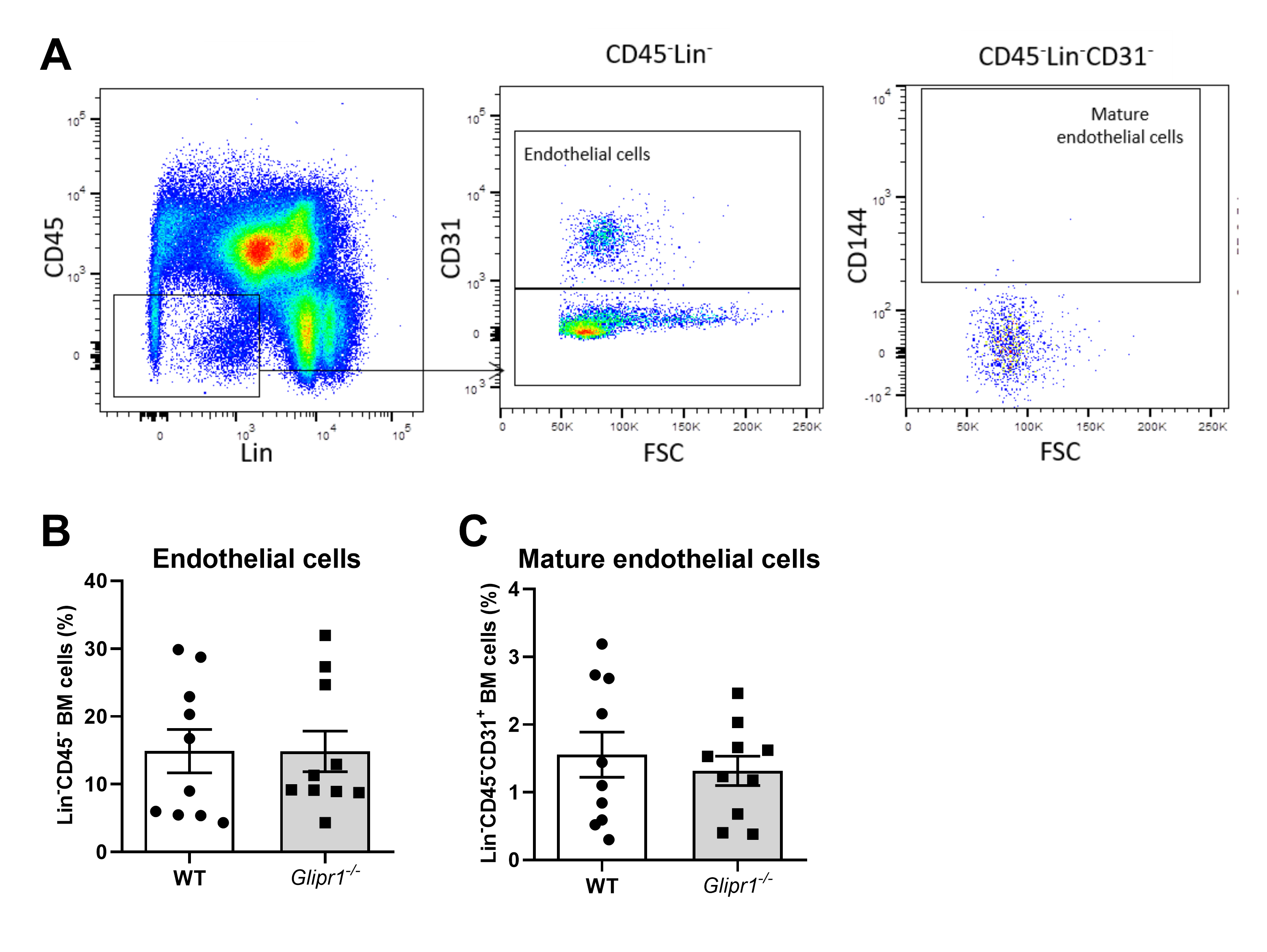

Supplement: S6 Fig — BM was collected from 12-month-old Glipr1-/- and WT control mice and single cell suspensions were prepared. The cells were stained with lineage markers, anti-CD11b, anti-CD45, anti-CD31 and anti-CD144 antibodies and analysed by flow cytometry. (A) Representative flow plots showing the gating strategy used to define total endothelial cells (Lin-CD45-CD31+) and mature endothelial cells (Lin-CD45-CD31+CD144+). Graphs show the percentage of endothelial cells (B) and mature endothelial cells (C) among Lin-CD45- BM cells. Graphs depict the mean ± SEM of n = 10 mice per genotype. (TIF) [file pone.0228408.s006.tif]

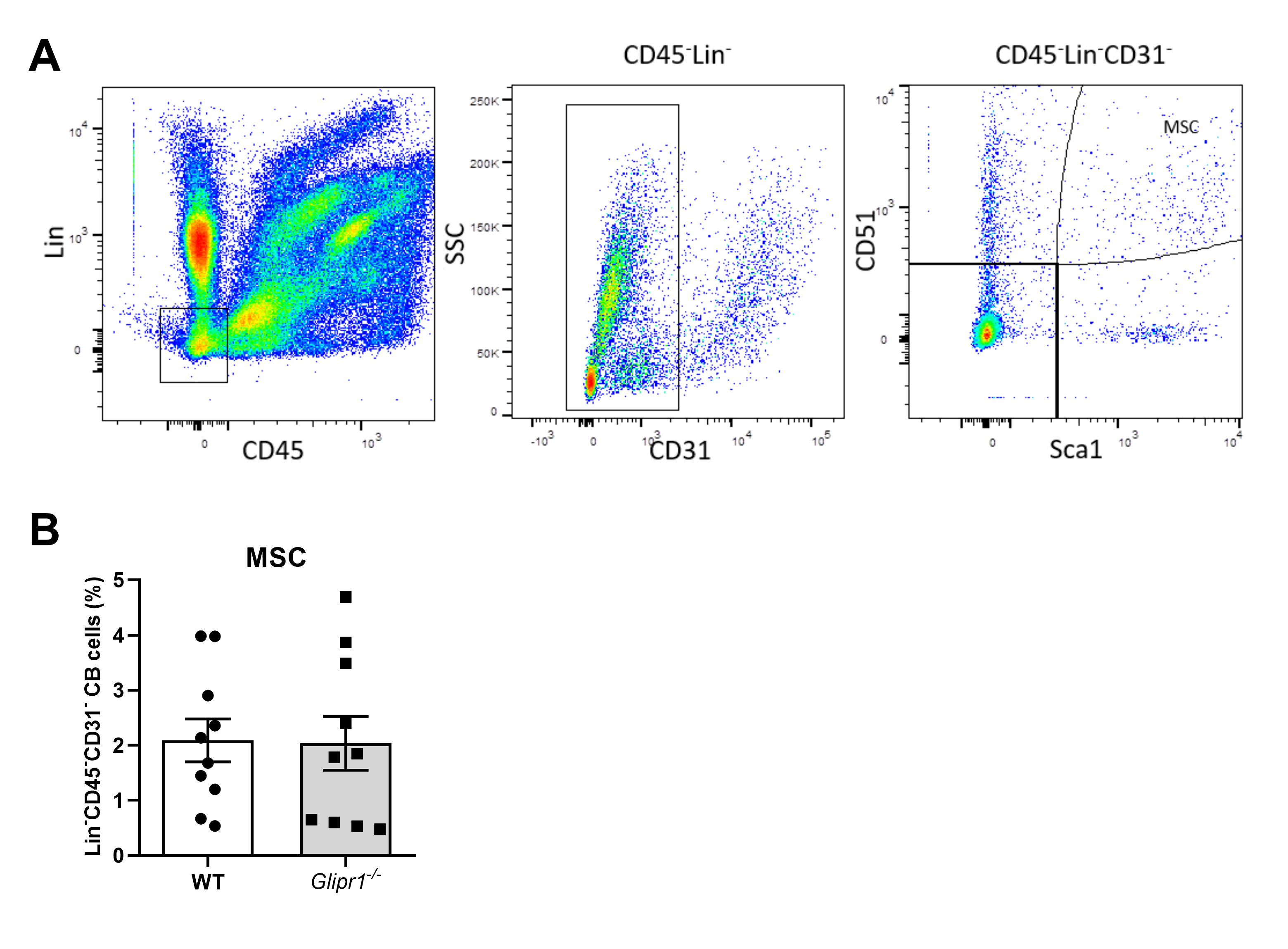

Supplement: S7 Fig — Compact bone (CB) was collected from 12-month-old Glipr1-/- and WT control mice and single cell suspensions were prepared. The cells were stained with lineage markers, anti-CD45, anti-CD31, anti-CD51 and anti-Sca1 antibodies and analysed by flow cytometry. (A) Representative flow plots showing the gating strategy used to define mesenchymal stem cells (MSCs; Lin-CD45-CD31-CD51-Sca1+). (B) Graph shows the percentage of MSCs among Lin-CD45-CD31- CB cells. Graph depicts the mean ± SEM of n = 10 mice per genotype. (TIF) [file pone.0228408.s007.tif]

Figure 2C

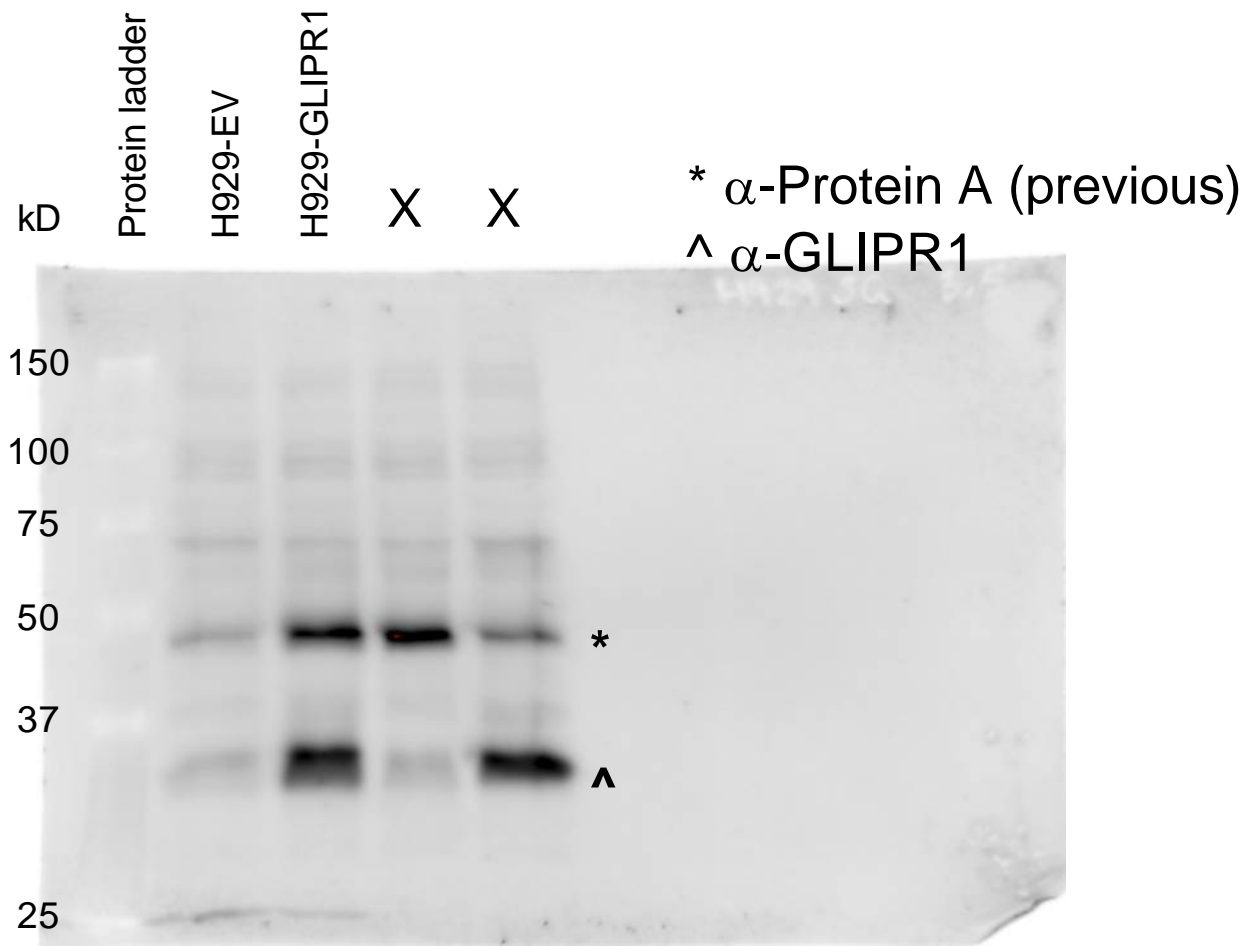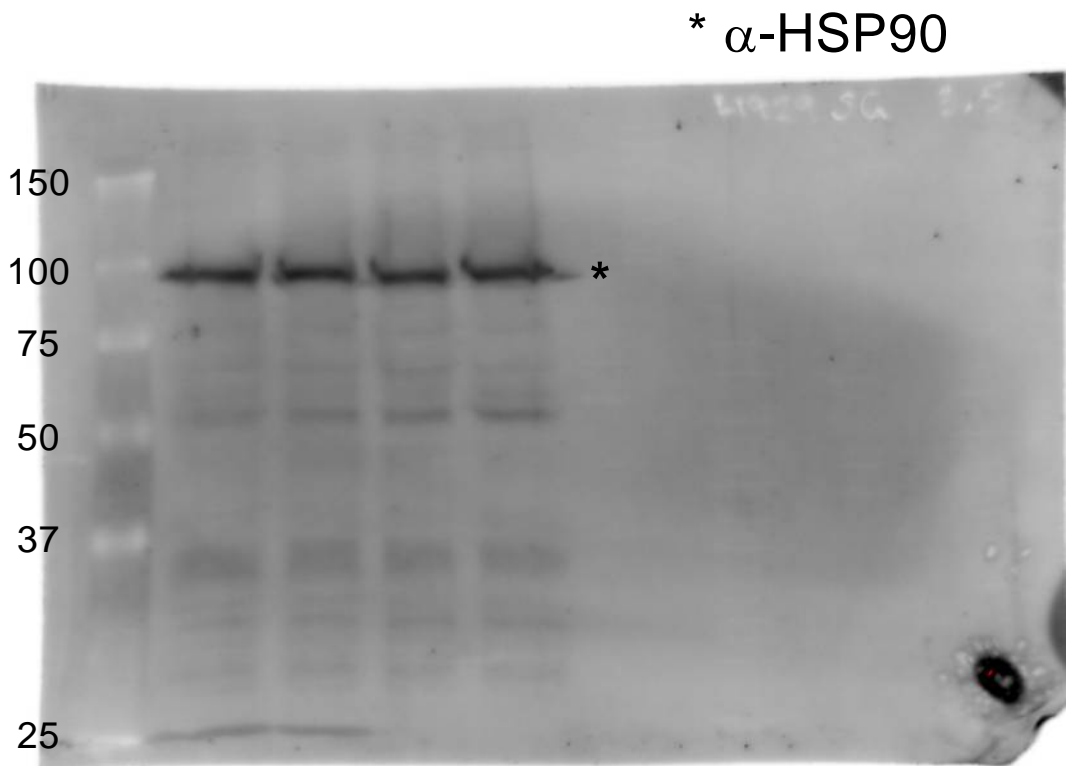

**Figure 3A**

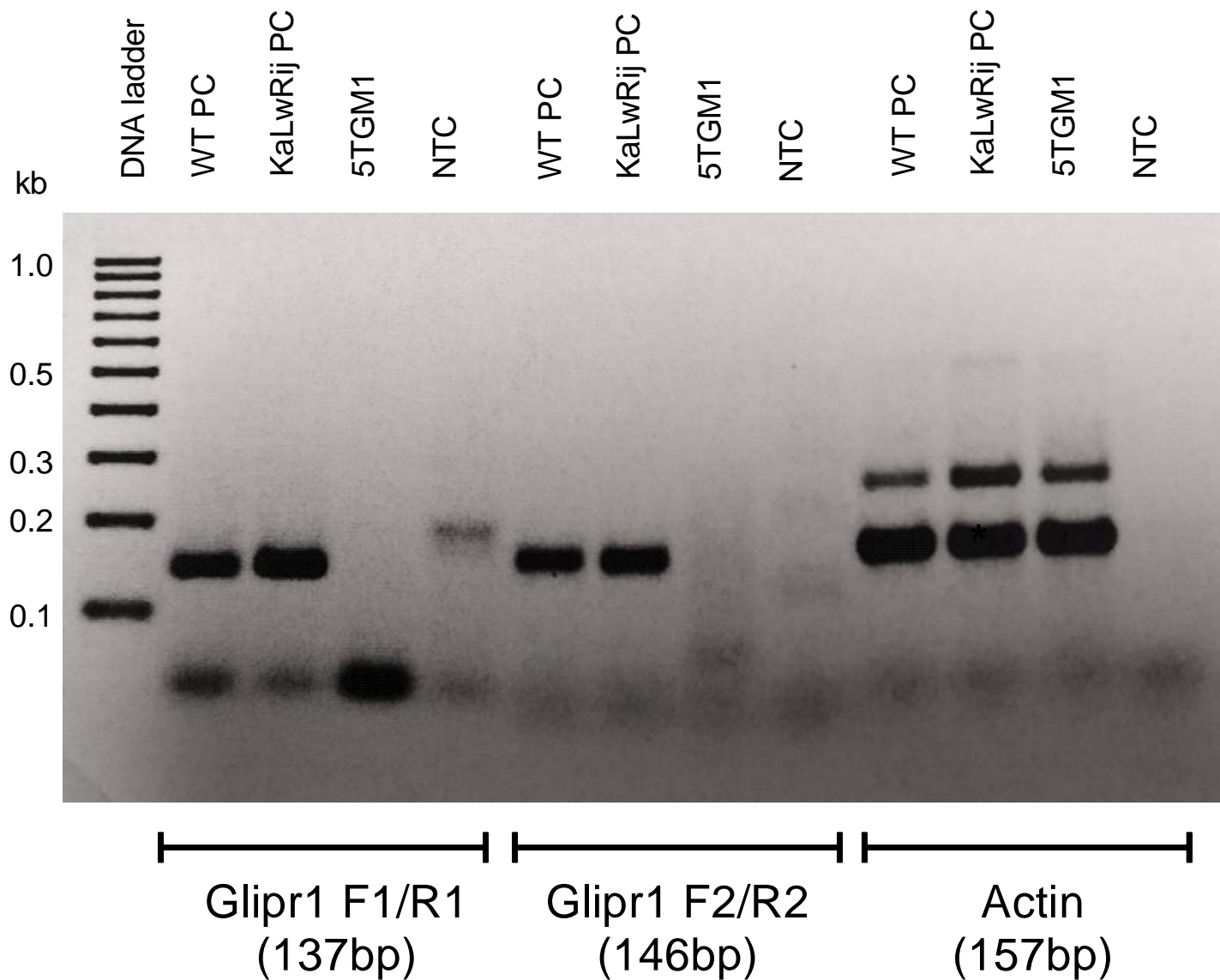

**Figure 3B**

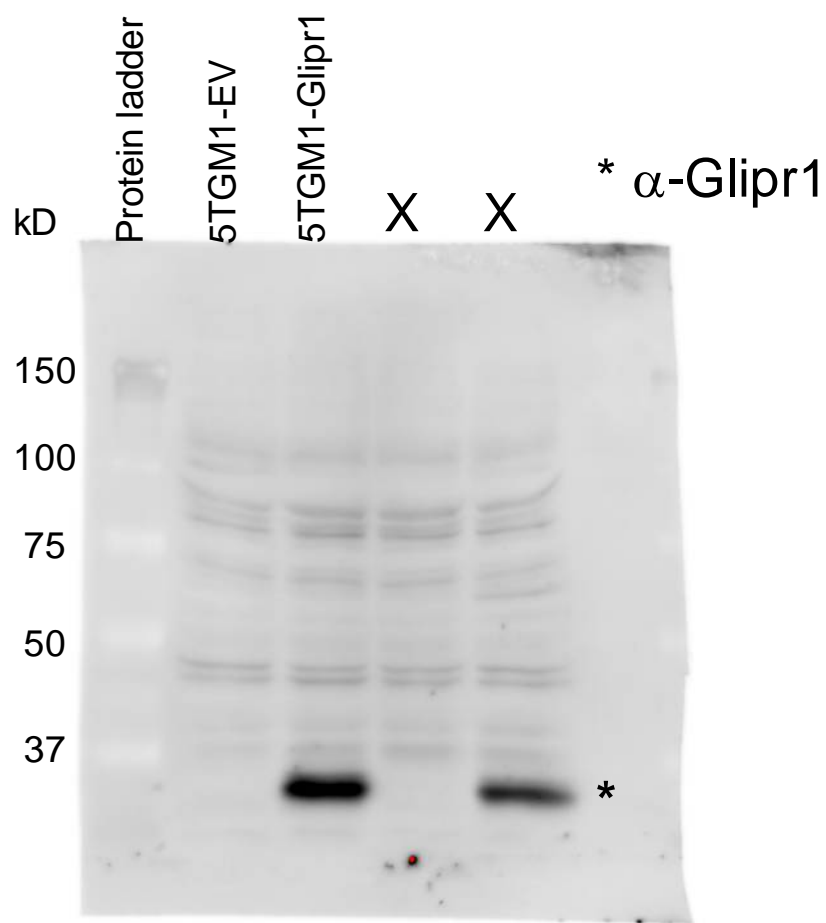

\*  $\alpha$ -Protein A (previous)

^  $\alpha$ -Hsp90

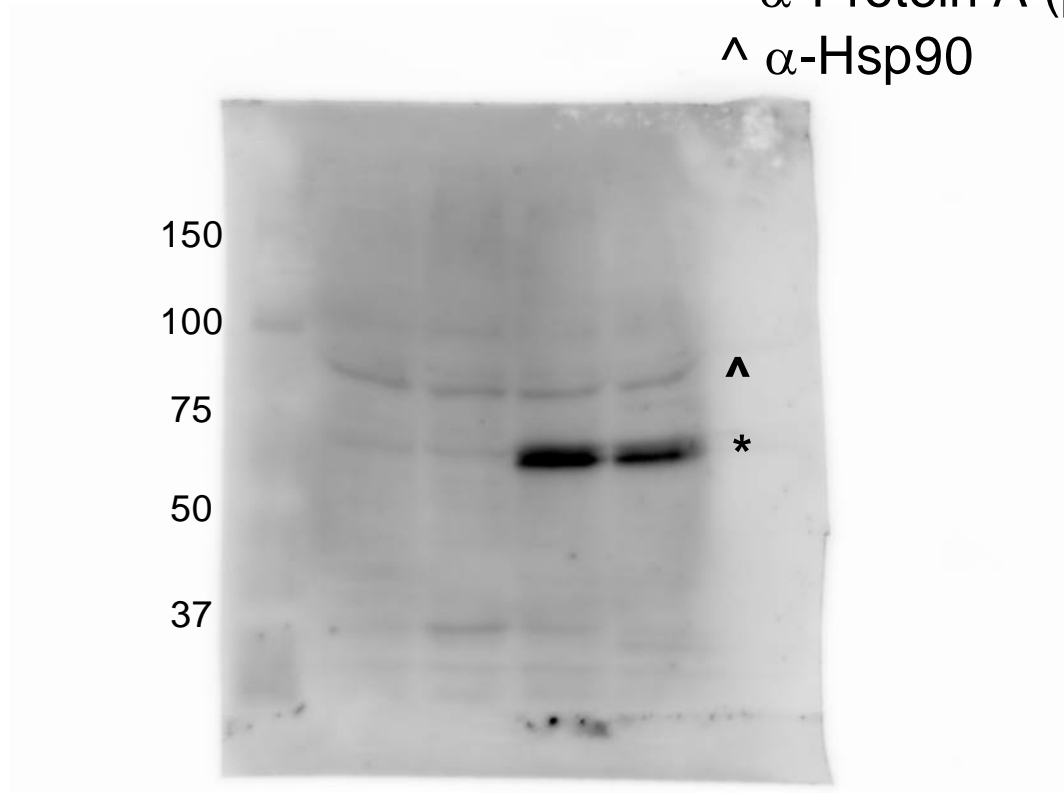

Figure 5D

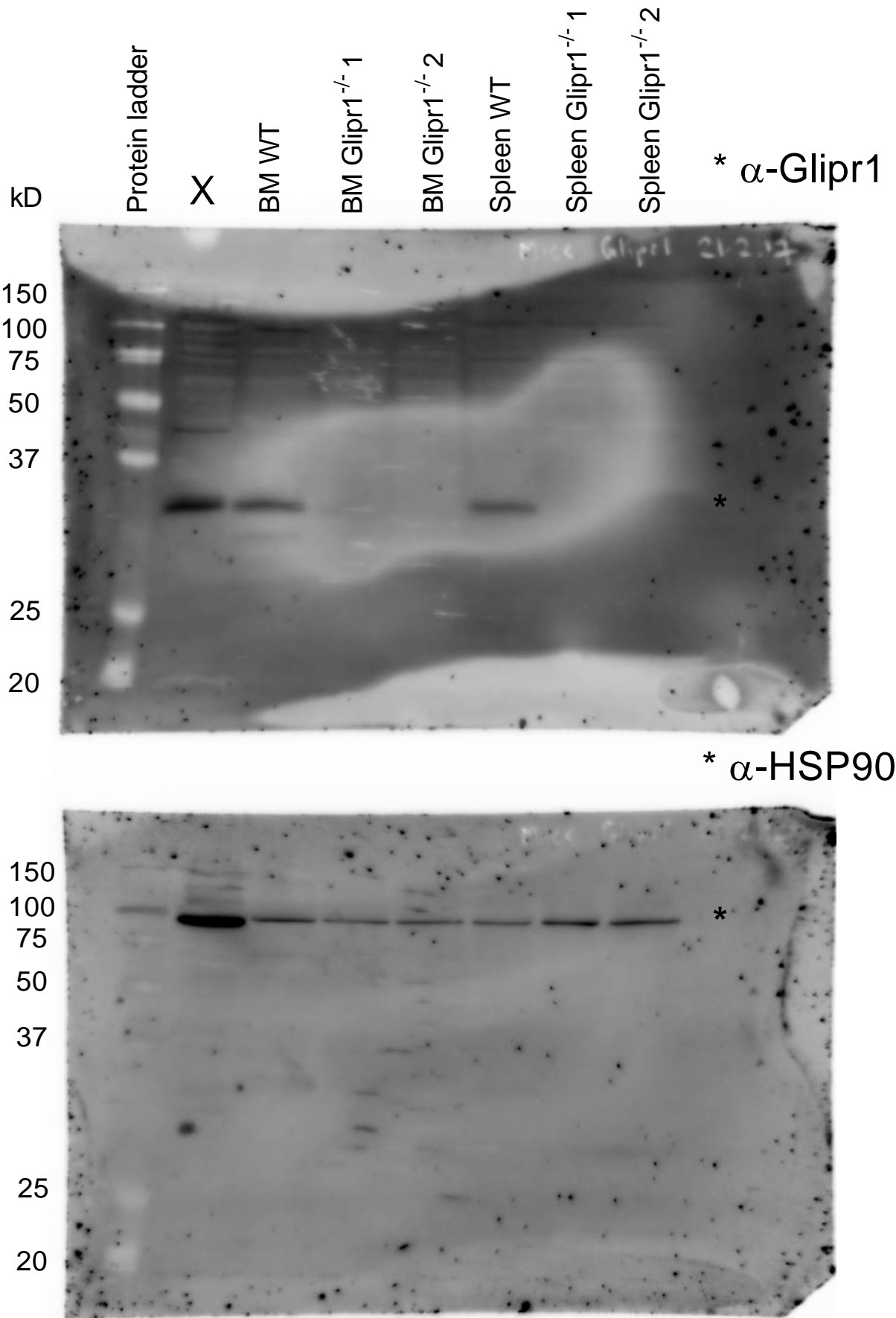

Supplement: S1 File — (PDF) [file pone.0228408.s010.pdf]
